# Changes in the combination of the triglyceride-glucose index and obesity indicators estimate the risk of cardiovascular disease

**DOI:** 10.1186/s12933-024-02281-4

**Published:** 2024-06-06

**Authors:** Xiaoqing Zhu, Weihao Xu, Tingting Song, Xinyan Wang, Qingsong Wang, Jun Li, Xixi Liu, Benchuan Hao, Tao Chen, Jun Guo

**Affiliations:** 1 Senior Department of Cardiology, The Sixth Medical Center of PLA General Hospital, Beijing, China; 2 Medical School of Chinese PLA, Beijing, China; 3 Department of Cardiology, Guangdong Provincial Cardiovascular Institute, Guangdong Provincial People’s Hospital, Guangdong Academy of Medical Sciences, Southern Medical University, Guangzhou, China; 4 Department of Geriatrics, Guangdong Provincial Geriatrics Institute, Guangdong Provincial People’s Hospital, Guangdong Academy of Medical Sciences, Southern Medical University, Guangzhou, China; 5 Haikou Cadre’s Sanitarium of Hainan Military Region, Haikou, China; 6 Department of Cardiology, The Second Medical Center, Chinese PLA General Hospital, Beijing, China

**Keywords:** Triglyceride-glucose index, Obesity indicators, Long-term change, Cardiovascular disease, K-means clustering

## Abstract

**Background:**

Cardiovascular disease (CVD) is closely associated with the triglyceride glucose (TyG) index and its related indicators, particularly its combination with obesity indices. However, there is limited research on the relationship between changes in TyG-related indices and CVD, as most studies have focused on baseline TyG-related indices.

**Methods:**

The data for this prospective cohort study were obtained from the China Health and Retirement Longitudinal Study. The exposures were changes in TyG-related indices and cumulative TyG-related indices from 2012 to 2015. The K-means algorithm was used to classify changes in each TyG-related index into four classes (Class 1 to Class 4). Multivariate logistic regressions were used to evaluate the associations between the changes in TyG-related indices and the incidence of CVD.

**Results:**

In total, 3243 participants were included in this study, of whom 1761 (54.4%) were female, with a mean age of 57.62 years at baseline. Over a 5-year follow-up, 637 (19.6%) participants developed CVD. Fully adjusted logistic regression analyses revealed significant positive associations between changes in TyG-related indices, cumulative TyG-related indices and the incidence of CVD. Among these changes in TyG-related indices, changes in TyG-waist circumference (WC) showed the strongest association with incident CVD. Compared to the participants in Class 1 of changes in TyG-WC, the odds ratio (OR) for participants in Class 2 was 1.41 (95% confidence interval (CI) 1.08–1.84), the OR for participants in Class 3 was 1.54 (95% CI 1.15–2.07), and the OR for participants in Class 4 was 1.94 (95% CI 1.34–2.80). Moreover, cumulative TyG-WC exhibited the strongest association with incident CVD among cumulative TyG-related indices. Compared to the participants in Quartile 1 of cumulative TyG-WC, the OR for participants in Quartile 2 was 1.33 (95% CI 1.00–1.76), the OR for participants in Quartile 3 was 1.46 (95% CI 1.09–1.96), and the OR for participants in Quartile 4 was 1.79 (95% CI 1.30–2.47).

**Conclusions:**

Changes in TyG-related indices are independently associated with the risk of CVD. Changes in TyG-WC are expected to become more effective indicators for identifying individuals at a heightened risk of CVD.

**Supplementary Information:**

The online version contains supplementary material available at 10.1186/s12933-024-02281-4.

## Introduction

Cardiovascular disease (CVD), a major global health concern, remains the leading cause of mortality and healthcare costs, imposing a substantial burden on the healthcare systems in China and worldwide [1–4]. Therefore, it is crucial to enhance current strategies for identifying individuals at high risk of CVD, thereby implementing targeted preventive measures to reduce the incidence of CVD and mortality.

Insulin resistance (IR), a hallmark of diabetes mellitus (DM), is characterized by a diminished tissue response to insulin [5–7]. Previous studies have indicated that IR may significantly contribute to the development of CVD [6, 8–10]. While the hyperinsulinemic-euglycemic clamp is considered the gold standard for assessing IR, its complex testing process constrains its clinical utility [11]. The triglyceride-glucose (TyG) index, a composite indicator of triglyceride (TG) and fasting plasma glucose (FBG) levels, has emerged as a cost-effective surrogate for IR [12, 13]. Recent studies have shown that the baseline TyG index, cumulative TyG index, and TyG index variability are associated with the incidence of CVD, coronary heart disease (CHD), myocardial infarction, stroke, and major adverse cardiovascular events [14–20].

Obesity is a growing global health concern that is strongly associated with IR and metabolic disorders [21]. Research indicates that combining the TyG index with obesity indicators, such as body mass index (BMI), waist circumference (WC), and waist height ratio (WHtR), may improve the accuracy of IR detection [13]. Previous studies have revealed that the combination of baseline TyG and obesity indicators can provide superior predictive value for CVD, CHD, stroke and hypertension when compared to TyG alone [18, 22–24]. A recent prospective cohort study indicated the significant relationship between changes in TyG-BMI and stroke incidence [25]. Moreover, cumulative TyG-BMI has also been demonstrated to be associated with CVD incidence [26].

While previous studies have extensively reported on the relationship between TyG and its related indices and CVD, there has been limited focus on the association between changes in TyG-related indices and CVD. To address this gap, this study aims to characterize and compare the predictive value of changes in TyG-BMI, TyG-WC, and TyG-WHtR for the incidence of CVD.

## Method

### Study participants

**Fig. 1:**
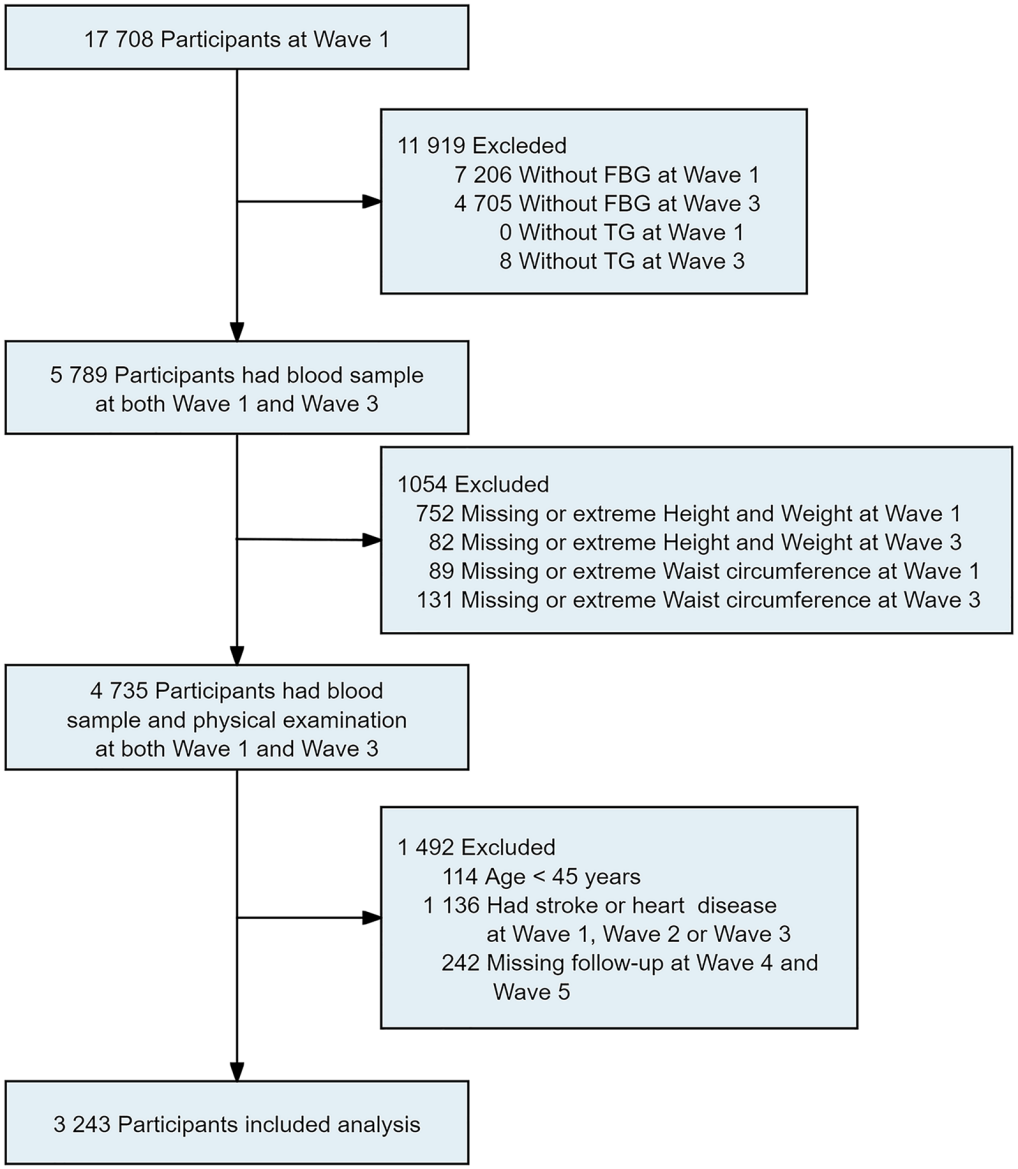
Flowchart of the inclusion and exclusion of participants. *FBG* fasting plasma glucose, *TG* triglyceride

The China Health and Retirement Longitudinal Study (CHARLS) is a national population-based longitudinal study [27]. In total, 17,708 participants were initially recruited nationwide in China from 2011 to 2012 (Wave 1). Participants were followed up every 2 to 3 years, with subsequent waves in 2013 (Wave 2), 2015 (Wave 3), 2018 (Wave 4), and 2020 (Wave 5). At each wave, the CHARLS interviewers conducted health-related surveys using the standardized questionnaire. Blood samples and physical measurements were collected at Waves 1 and 3. Initially, we included 5789 participants with complete FBG and TG at Waves 1 and 3, and then excluded participants without height, weight, or WC at Waves 1 and 3 (n = 1,54). Participants who were younger than 45 years old (n = 114), had prevalent CVD at Waves 1, 2, or 3 (n = 1136), or had missing follow-up data at Waves 4 and 5 (n = 242) were also excluded. Ultimately, 3243 participants were enrolled in our study (Fig. 1). All participants provided informed consent, and CHARLS received approval from the institutional review board of Peking University.

### Assessment of exposures

The exposures were changes in TyG-related indices and cumulative TyG-related indices over the period from 2012 to 2015. TyG-related indices were calculated as follows [13, 14, 24]:

$$ TyG - WC = \ln \left( {\frac{{TG \left( {{\text{mg}}/{\text{dl}}} \right) \times FBG \left( {{\text{mg}}/{\text{dl}}} \right)}}{2}} \right) \times WC \left( {{\text{cm}}} \right); $$

$$ TyG - BMI = \ln \left( {\frac{{TG \left( {{\text{mg}}/{\text{dl}}} \right) \times FBG \left( {{\text{mg}}/{\text{dl}}} \right)}}{2}} \right) \times BMI \left( {{\text{kg}}/{\text{m}}^{2} } \right); $$

$$ TyG - WHtR = \ln \left( {\frac{{TG \left( {{\text{mg}}/{\text{dl}}} \right) \times FBG \left( {{\text{mg}}/{\text{dl}}} \right)}}{2}} \right) \times WHtR. $$

Cumulative TyG-WC, TyG-BMI, and TyG-WHtR, were calculated using the formulas below [16, 25]: (TyG-WC_2012_ + TyG-WC_2015_)/2 × time(2015 − 2012); (TyG-BMI_2012_ + TyG-BMI_2015_)/2 × time(2015 − 2012); (TyG-WHtR_2012_ + TyG-WHtR _2015_)/2 × time(2015 − 2012). Height, weight and WC were measured by trained nurses. BMI was calculated as weight in kilograms divided by height in metres squared. WHtR was calculated as WC in centimetres divided by height in centimetres.

**Fig. 2:**
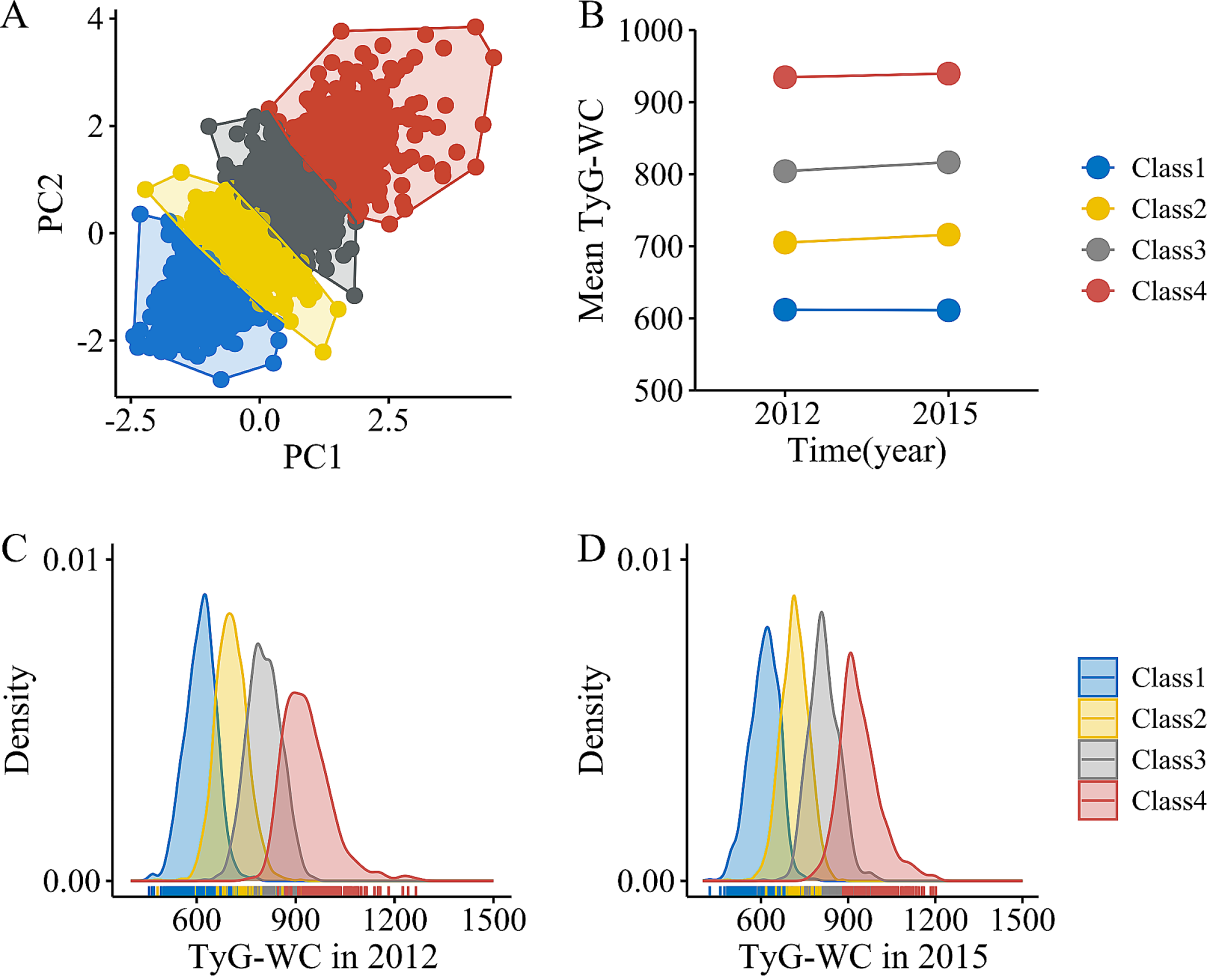
Clustering of changes in TyG-WC from Wave 1 to Wave 3. Changes in TyG-WC classified into four classes using the K‑means algorithm (**A**); Mean TyG-WC for the four classes in 2012 and 2015 (**B**); Distribution of TyG‑WC in 2012 or 2015 (**C**, **D**). *PC* principal component, *TyG* triglyceride‑glucose index, *WC* waist circumference

Changes in TyG-WC, TyG-BMI, and TyG-WHtR from Wave 1 to Wave 3 were respectively classified into four classes (Class 1 to Class 4) using the K-means clustering algorithm (Figs. 2, S1 and S2). Specifically, Class 1 represented consistently low TyG-related indices, Class 2 indicated moderate TyG-related indices with a slight increasing trend, Class 3 denoted high TyG-related indices with a slight increasing trend, and Class 4 was characterized by consistently highest TyG-related indices. The mechanism of the K-means algorithm has been described in detail before [28, 29]. A detailed description of the clustering is shown in Additional file.

### Assessment of incident CVD

The primary outcome was incident CVD, which was ascertained by responses to the following questions: “Have you been diagnosed with stroke or heart condition (including heart attack, coronary heart disease, angina, congestive heart failure, or other heart problems) by a doctor?” or the question “Are you now undergoing any treatments to treat stroke or heart condition?”.

### Covariates and missing data handling

The following covariates were collected: (1) sociodemographic factors: age, sex, education level, and marital status; (2) lifestyle factors: smoking status and drinking status; (3) medical history: hypertension, DM, dyslipidaemia, and kidney disease; (4) physical measurements: weight, height, BMI, WC, WHtR, systolic blood pressure (SBP), and diastolic blood pressure (DBP); and (5) laboratory examinations: total cholesterol (TC), high-density lipoprotein cholesterol (HDL-C), low-density lipoprotein cholesterol (LDL-C), TG, FBG, estimated glomerular filtration rate (eGFR), glycosylated haemoglobin (HbA1c) and hypersensitive C-reactive protein (hsCRP). The eGFR was calculated using the modified Modification of Diet in Renal Disease (MDRD) equation [30]: *eGFR* = {175 × *Scr* (mg/dl)^−1.234^ × *Age*^−0.179^ × 0.79 (if female). DM was defined as self-reported DM, any treatment for DM, or FBG ≥ 126 mg/dl. Hypertension was defined as self-reported hypertension, any treatment for hypertension, SBP ≥ 140 mmHg, or DBP ≥ 90 mmHg. Participants were considered to have dyslipidaemia if they self-reported a history of dyslipidaemia, were undergoing treatment for dyslipidaemia, or had TC ≥ 240 mg/dl, TllG ≥ 150 mg/dL, LDL-C ≥ 160 mg/dl, or HDL-C < 40 mg/dl. To address missing data, we utilized multiple imputation of chained equations, generating five imputed datasets to ensure robustness in our analyses.

### Statistical analysis

Baseline data were expressed as mean ± standard deviation (SD) or median with interquartile range (IQR) for continuous variables and as number (percentage or frequency) for categorical variables. Descriptive analyses were conducted using the χ^2^ test, one-way analysis of variance test (ANOVA) or Kruskal–Wallis test, as appropriate.

Logistic regression models were used to assess odd ratios (ORs) and 95% confidence intervals (CIs) for the associations between changes in TyG-related indices and the incidence of CVD. Four multivariate models were constructed: Model 1 adjusted for age and sex; Model 2 adjusted for age, sex, marital status, educational level, and smoking and drinking status; Model 3 further adjusted for history of hypertension, DM, dyslipidaemia, kidney disease, treatment for hypertension, DM, dyslipidaemia, SBP, and DBP based on Model 2; Model 4 adjusted for variables in Model 3 and TC, HDL-C, LDL-C, HbA1c, the eGFR, and hsCRP. C-statistics were calculated to compare the predictive ability of changes in TyG and its related indices. Additionally, sensitivity analyses were performed by repeating the primary analyses using the complete dataset without imputation.

All statistical analyses were performed using R version 4.2.2 (R Foundation for Statistical Computing, Vienna, Austria). A two-sided *p*-value of < 0.05 was considered statistically significant.

## Results

### Baseline characteristics

The characteristics of the 3243 participants according to classes of changes in TyG-WC, TyG-BMI, and TyG-WHtR are presented in Table 1 and Additional file (Tables S1 and S2). Among the participants, 45.6% (n = 1477) were men, with a mean age at baseline of 57.62 ± 8.40 years. Compared with those in Class 1 of changes in TyG-WC, participants in the other classes were more likely to be younger, female, nonsmokers, non-drinkers. They also had a higher prevalence of hypertension, DM and dyslipidaemia, higher SBP, DBP, TC, LDL-C, and HbA1c levels, and lower levels of HDL-C.

**Table 1:** Baseline characteristics of 3243 participants according to TyG-WC change classes

Characteristic	Overall (n = 3243)	Changes in TyG-WC				
		Class 1 (n = 825)	Class 2 (n = 1106)	Class 3 (n = 882)	Class 4 (n = 430)	P value
Age, mean ± SD, years	57.62 ± 8.40	58.29 ± 8.69	57.59 ± 8.49	57.31 ± 8.24	57.07 ± 7.85	0.04
Sex						< 0.001
Female	1761 (54.4%)	371 (45.1%)	613 (55.5%)	519 (58.8%)	258 (60.1%)	
Male	1477 (45.6%)	452 (54.9%)	491 (44.5%)	363 (41.2%)	171 (39.9%)	
Marital status						0.125
Married	2810 (86.6%)	705 (85.5%)	946 (85.5%)	776 (88%)	383 (89.1%)	
Others	433 (13.4%)	120 (14.5%)	160 (14.5%)	106 (12%)	47 (10.9%)	
Education level						0.244
No formal education	927 (28.6%)	233 (28.2%)	336 (30.4%)	234 (26.5%)	124 (28.8%)	
Primary school	1340 (41.3%)	365 (44.2%)	450 (40.7%)	355 (40.2%)	170 (39.5%)	
Middle or high school	896 (27.6%)	212 (25.7%)	293 (26.5%)	267 (30.3%)	124 (28.8%)	
College or above	80 (2.5%)	15 (1.8%)	27 (2.4%)	26 (2.9%)	12 (2.8%)	
Smoking status^a^						< 0.001
Never	2026 (62.6%)	434 (52.8%)	709 (64.2%)	585 (66.6%)	298 (69.5%)	
Former	225 (7.0%)	47 (5.7%)	74 (6.7%)	67 (7.6%)	37 (8.6%)	
Current	983 (30.4%)	341 (41.5%)	321 (29.1%)	227 (25.8%)	94 (21.9%)	
Drinking status^a^						< 0.001
Never	1890 (58.4%)	430 (52.3%)	660 (59.7%)	524 (59.5%)	276 (64.2%)	
Former	230 (7.1%)	69 (8.4%)	63 (5.7%)	64 (7.3%)	34 (7.9%)	
Current	1117 (34.5%)	323 (39.3%)	382 (34.6%)	292 (33.2%)	120 (27.9%)	
Hypertension^a^						< 0.001
No	2066 (64.1%)	645 (78.9%)	758 (68.8%)	478 (54.6%)	185 (43.1%)	
Yes	1159 (35.9%)	173 (21.1%)	344 (31.2%)	398 (45.4%)	244 (56.9%)	
Diabetes^a^						< 0.001
No	2801 (87.1%)	767 (93.5%)	1015 (92.3%)	740 (85%)	279 (65.5%)	
Yes	416 (12.9%)	53 (6.5%)	85 (7.7%)	131 (15%)	147 (34.5%)	
Dyslipidaemia^a^						< 0.001
No	1694 (52.9%)	640 (78.6%)	666 (61.2%)	325 (37.3%)	63 (14.8%)	
Yes	1507 (47.1%)	174 (21.4%)	423 (38.8%)	547 (62.7%)	363 (85.2%)	
Kidney disease^a^						0.278
No	3048 (94.8%)	766 (93.9%)	1039 (94.5%)	832 (95.3%)	411 (96.3%)	
Yes	167 (5.2%)	50 (6.1%)	60 (5.5%)	41 (4.7%)	16 (3.7%)	
Systolic blood pressure, mean ± SD, mmHg^a^	128.58 ± 20.37	122.30 ± 18.81	126.99 ± 19.40	132.52 ± 20.59	136.73 ± 20.84	< 0.001
Diastolic blood pressure, mean ± SD, mmHg^a^	75.29 ± 11.77	71.23 ± 10.78	74.46 ± 11.42	77.59 ± 11.41	80.51 ± 12.24	< 0.001
TC, mean ± SD, mg/dl	194.12 ± 38.74	183.77 ± 35.40	192.43 ± 36.85	198.06 ± 36.53	210.21 ± 46.80	< 0.001
HDL-C, mean ± SD, mg/dl	51.30 ± 15.15	60.11 ± 16.47	52.98 ± 13.32	46.31 ± 12.03	40.32 ± 11.43	< 0.001
LDL-C, mean ± SD, mg/dl^a^	117.15 ± 34.55	109.49 ± 29.95	119.34 ± 33.51	121.69 ± 34.70	116.92 ± 42.09	< 0.001
HbA1c, mean ± SD, %	5.27 ± 0.82	5.11 ± 0.55	5.18 ± 0.64	5.30 ± 0.82	5.77 ± 1.32	< 0.001
eGFR, mean ± SD, ml/min/1.73 m^2a^	110.77 ± 29.11	112.19 ± 28.52	110.75 ± 26.55	109.19 ± 29.77	111.31 ± 34.58	0.194
TyG_2012_, mean ± SD	8.66 ± 0.66	8.19 ± 0.42	8.51 ± 0.47	8.88 ± 0.54	9.46 ± 0.74	< 0.001
TyG_2015_, mean ± SD	8.66 ± 0.61	8.18 ± 0.38	8.53 ± 0.43	8.91 ± 0.51	9.43 ± 0.58	< 0.001
BMI_2012_, mean ± SD	23.51 ± 3.70	20.25 ± 2.16	22.73 ± 2.76	25.36 ± 2.83	27.99 ± 3.09	< 0.001
BMI_2015_, mean ± SD	23.69 ± 3.55	20.34 ± 2.33	23.00 ± 2.23	25.47 ± 2.41	28.22 ± 3.20	< 0.001
WC_2012_, mean ± SD	85.13 ± 9.64	74.79 ± 5.20	82.93 ± 5.23	90.78 ± 5.54	99.03 ± 6.33	< 0.001
WC_2015_, mean ± SD	85.90 ± 10.06	74.75 ± 5.66	84.08 ± 5.36	91.84 ± 5.60	99.78 ± 7.22	< 0.001
WHtR_2012_, mean ± SD	0.54 ± 0.06	0.48 ± 0.04	0.53 ± 0.04	0.57 ± 0.04	0.62 ± 0.05	< 0.001
WHtR_2015_, mean ± SD	0.55 ± 0.07	0.48 ± 0.04	0.54 ± 0.04	0.58 ± 0.04	0.63 ± 0.05	< 0.001
TyG-BMI_2012_, mean ± SD	204.18 ± 39.55	165.72 ± 18.51	193.25 ± 23.28	224.71 ± 25.21	263.98 ± 29.26	< 0.001
TyG-BMI_2015_, mean ± SD	205.96 ± 38.66	166.33 ± 19.95	195.96 ± 19.83	226.49 ± 21.70	265.63 ± 28.92	< 0.001
TyG-WC_2012_, mean ± SD	738.92 ± 116.04	612.01 ± 46.65	705.25 ± 47.28	804.38 ± 50.74	934.77 ± 73.38	< 0.001
TyG-WC_2015_, mean ± SD	746.43 ± 118.41	611.21 ± 49.80	716.15 ± 47.03	816.65 ± 51.72	939.72 ± 69.97	< 0.001
TyG-WHtR_2012_, mean ± SD	4.69 ± 0.75	3.90 ± 0.34	4.48 ± 0.37	5.09 ± 0.42	5.88 ± 0.52	< 0.001
TyG-WHtR_2015_, mean ± SD	4.75 ± 0.78	3.91 ± 0.37	4.57 ± 0.40	5.18 ± 0.42	5.93 ± 0.50	< 0.001
Cumulative TyG-BMI, mean ± SD	615.2 ± 111.8	498.1 ± 51.2	583.8 ± 54.7	676.8 ± 58.3	794.4 ± 76.7	< 0.001
Cumulative TyG-WC, mean ± SD	2228.0 ± 332.6	1834.8 ± 108.4	2132.1 ± 85.5	2431.5 ± 97.2	2811.7 ± 165.9	< 0.001
Cumulative TyG-WHtR, mean ± SD	14.15 ± 2.17	11.72 ± 0.89	13.57 ± 0.90	15.41 ± 1.00	17.72 ± 1.26	< 0.001

*BMI* body mass index, *WC* waist circumference, *WHtR* waist height ratio, *eGFR* estimated glomerular filtration ratio, *HbA1c* glycated haemoglobin, *HDL-C* high-density lipoprotein cholesterol, *LDL-C* low-density lipoprotein cholesterol, *SD* standard deviation, *TC* total cholesterol, *TyG* triglyceride-glucose index

^a^Missing data: 5 for sex, 9 for smoking status, 6 for drinking status, 18 for hypertension, 26 for diabetes, 42 for dyslipidaemia, 28 for kidney disease, 13 for systolic blood pressure, 13 for diastolic blood pressure, 7 for LDL-C, 1 for the eGFR, and 11 for HbA1c

### Associations between changes in TyG-WC index and the incidence of CVD

During a 5-year follow-up, a total of 637 (19.6%) participants developed CVD events. Table 2 reveals the associations between changes in the TyG-WC index and the incidence of CVD. After adjustment for covariates in Model 4, participants in Class 2 (OR = 1.41, 95% CI 1.08–1.84), Class 3 (OR = 1.54, 95% CI 1.15–2.07), and Class 4 (OR = 1.94, 95% CI 1.34–2.80) of changes in TyG-WC exhibited a greater risk of CVD compared to those in Class 1. Moreover, participants in Quartile 2 (OR = 1.33, 95% CI 1.00–1.76), Quartile 3 (OR = 1.46, 95% CI 1.09–1.96), and Quartile 4 (OR = 1.79, 95% CI 1.30–2.47) showed an increased risk of CVD compared to those in Quartile 1 of cumulative TyG-WC.

**Table 2:** Associations of changes in TyG-WC and cumulative TyG-WC with cardiovascular disease incidence

	Event/total	Model 1^a^		Model 2^b^		Model 3^c^		Model 4^d^	
		OR (95%CI)	P value	OR (95%CI)	P value	OR (95%CI)	P value	OR (95%CI)	P value
Changes in TyG-WC									
Class1	108/825	Reference		Reference		Reference		Reference	
Class2	209/1106	1.56 (1.21–2.01)	0.001	1.58 (1.22–2.03)	< 0.001	1.45 (1.11–1.88)	0.005	1.41 (1.08–1.84)	0.012
Class3	196/882	1.93 (1.48–2.5)	< 0.001	1.94 (1.49–2.52)	< 0.001	1.56 (1.18–2.07)	0.002	1.54 (1.15–2.07)	0.004
Class4	124/430	2.76 (2.06–3.7)	< 0.001	2.77 (2.06–3.73)	< 0.001	1.94 (1.38–2.73)	< 0.001	1.94 (1.34–2.80)	< 0.001
Cumulative TyG-WC									
Quartile1	107/811	Reference		Reference		Reference		Reference	
Quartile2	145/810	1.45 (1.10–1.90)	0.008	1.46 (1.11–1.92)	0.007	1.36 (1.03–1.79)	0.030	1.33 (1.00–1.76)	0.047
Quartile3	168/811	1.74 (1.33–2.28)	< 0.001	1.76 (1.34–2.30)	< 0.001	1.50 (1.13–1.99)	0.005	1.46 (1.09–1.96)	0.011
Quartile4	217/811	2.46 (1.89–3.18)	< 0.001	2.47 (1.90–3.20)	< 0.001	1.80 (1.34–2.44)	< 0.001	1.79 (1.30–2.47)	< 0.001
P for trend	637/3243	1.33 (1.23–1.44)	< 0.001	1.33 (1.23–1.45)	< 0.001	1.20 (1.09–1.32)	< 0.001	1.20 (1.08–1.33)	0.001

*WC* waist circumference, *CI* confidence interval, *OR* odds ratio, *TyG* triglyceride-glucose index

^a^Adjusted for age and sex

^b^Adjusted for age, sex, marital status, educational level, smoking status, and drinking status

^c^Adjusted for variables in Model 2 and history of hypertension, diabetes, dyslipidaemia, kidney disease, medication use for hypertension, medication use for diabetes, medication use for dyslipidaemia, systolic blood pressure, diastolic blood pressure

^d^Adjusted for variables in Model 3 and total cholesterol, HDL-C, LDL-C, HbA1c, the eGFR, and hsCRP

### Associations between changes in TyG-BMI index and incident CVD

Changes in TyG-BMI were associated with incident CVD after fully adjusting for covariates (Table S3). Specifically, participants in Class 2 (OR = 1.32, 95% CI 1.03–1.68) and Class 4 (OR = 1.68, 95% CI 1.15–2.46) of changes in TyG-BMI had a higher risk of CVD compared to those in Class 1, while the increase in risk was not significant for Class 3 (OR = 1.22, 95% CI 0.91–1.64). Furthermore, compared to Quartile 1, participants in Quartile 4 of cumulative TyG-BMI showed an elevated risk of CVD (OR = 1.49, 95% CI 1.09–2.05), whereas the risk increase was not significant for Quartile 2 (OR = 1.28, 95% CI 0.98–1.69) and Quartile 3 (OR = 1.26, 95% CI 0.94–1.68).

### Associations between changes in TyG-WHtR index and incident CVD

The associations between changes in TyG-WHtR index and the incidence of CVD are presented in the Table S4. After adjustment for potential confounders, the risk of CVD was significantly higher in Class 4 (OR = 1.53; 95% CI 1.05–2.24) compared to Class 1, but insignificantly increased in Class 2 (OR = 1.23; 95% CI 0.95–1.59) and Class 3 (OR = 1.25; 95% CI 0.93–1.68). Similarly, only the highest quartile of cumulative TyG-WHtR (OR = 1.47; 95% CI 1.05–2.04) showed a significant increase in CVD risk compared to Quartile 1, while the risk increase was not significant for Quartile 2 (OR = 1.23; 95% CI 0.93–1.63) and Quartile 3 (OR = 1.32; 95% CI 0.98–1.78) relative to Quartile 1.

### Comparison of the predictive value of changes in TyG and its related indices

Additionally, we examined the associations between changes in TyG, cumulative TyG, and incident CVD (Table S5). Table 3 displays the comparison of the predictive abilities of changes in TyG and its related indices for CVD. Results indicated that both changes in TyG-WC (C-statistic = 0.584, 95% CI 0.561–0.608) and cumulative TyG-WC (C-statistic = 0.586, 95% CI 0.542–0.610) demonstrated superior discrimination for CVD among these indicators. Changes in TyG-WC significantly outperformed changes in TyG alone (∆C-statistic = 0.022, 95% CI 0.003–0.041). Similarly, cumulative TyG-WC displayed a significant increase in the ∆C-statistic compared to cumulative TyG alone (∆C-statistic = 0.026, 95% CI 0.007–0.046). Changes in TyG-BMI and TyG-WHtR did not exhibit statistically significant differences in C-statistics when compared to changes in TyG alone. Moreover, there were no statistically significant differences in C-statistics between cumulative TyG-BMI, TyG-WHtR, and TyG alone.

**Table 3:** Comparison of the predictive value of changes in TyG-WC, TyG-BMI, TyG-WHtR, and TyG for incident CVD

	C-statistic^a^	C-statistic^b^	ΔC-statistic^c^	P-value for ΔC
Changes in				
TyG-WC vs. TyG	0.584(0.561–0.608)	0.562(0.538–0.586)	0.022(0.003–0.041)	0.022
TyG-BMI vs. TyG	0.565(0.541–0.588)	0.562(0.538–0.586)	0.003(-0.021–0.023)	0.823
TyG-WHtR vs. TyG	0.578(0.554–0.601)	0.562(0.538–0.586)	0.015(-0.006–0.035)	0.134
Cumulative				
TyG-WC vs. TyG	0.586(0.542–0.610)	0.559(0.536–0.583)	0.026(0.007–0.046)	0.007
TyG-BMI vs. TyG	0.566(0.542–0.590)	0.559(0.536–0.583)	0.007(-0.017–0.028)	0.551
TyG-WHtR vs. TyG	0.579(0.556–0.603)	0.559(0.536–0.583)	0.020(-0.001–0.040)	0.056

*TyG* triglyceride‑glucose index, *WC* waist circumference, *BMI* body mass index, *WHtR* waist height ratio

^a^C-statistic of the first indicator

^b^C-statistic of the second indicator

^c^ΔC-statistic = C-statistic^a^ − C-statistic^b^

### Incremental predictive value of changes in TyG-related indices

We further calculated and compared the C-statistics to assess the incremental predictive values of changes in TyG-obesity indices beyond the baseline obesity indicators, TG, FBG, or TyG-obesity indices. Results were displayed in Tables S6, S7, S8.

### Sensitivity analyses

Sensitivity analyses performed using the complete dataset without imputation yielded consistent results with the primary analysis (Tables S9, S10, S11). Changes in TyG-WC and cumulative TyG-WC consistently showed the strongest association with the incidence of CVD.

## Discussion

Our study revealed that changes in TyG-WC, TyG-BMI, and TyG-WHtR, as well as the cumulative TyG-WC, TyG-BMI, and TyG-WHtR, were independently associated with incident CVD in participants from the CHARLS cohort. Notably, among these change patterns of TyG-related indices, changes in TyG-WC had the strongest association with CVD incidence. Furthermore, the cumulative TyG-WC demonstrated a stronger correlation with incident CVD compared to the cumulative TyG-BMI and TyG-WHtR. These trends remained steady in subsequent sensitivity analyses.

Previous investigations have provided evidence demonstrating that the TyG and its related indices are reliable predictors of IR [13, 31, 32]. These indices have gained popularity in recent studies as they are derived from easily available measurements of FBG, TG, and obesity indicators. Some studies have suggested the underlying biological mechanism of TyG-related indices in the development of IR. Pathological conditions such as hyperlipidaemia and/or hyperglycaemia can enhance the formation of Advanced Glycation End Products (AGEs) in the human body [33, 34]. The accumulation of AGEs in metabolic organs can promote oxidative stress, inflammation and the development of IR [35, 36]. Additionally, adipose tissue has been demonstrated to be the primary source of oxidative stress [37]. Enlarged adipose tissues can release toxic lipids, disrupting insulin sensitivity modulation and further contributing to the development of IR [21]. Consequently, adiposity indices including BMI, WC, and WHtR may provide incremental value for predicting IR when combined with TyG. Two cross-sectional studies have indicated that TyG-WC, TyG-WHtR, and TyG-BMI were more effective than TyG alone in detecting IR [32]. Moreover, findings from a cross-sectional survey within the National Health and Nutrition Examination Survey (NHANES) dataset, which included 9884 participants, suggested that TyG-WC may be a more sensitive index for assessing IR compared to other indicators [31].

Previous studies have validated the associations between TyG-related indices and stroke, hypertension, atrial fibrillation recurrence, and carotid atherosclerosis [24, 38–40]. Additionally, baseline TyG-related indices have been identified as useful predictors for CVD and CHD [18, 22–24]. Recent studies have also revealed the association between TyG-related indices and incident CVD [18, 23]. However, these studies have a limitation in that they only assessed TyG-related indices at baseline. Only one study has explored the association between cumulative TyG-BMI and the risk of CVD [26]. An analysis of the Coronary Artery Risk Development in Young Adults study, which included 4754 participants in the analysis of the trajectory of TyG and incident CVD, revealed that a higher long-term trajectory of TyG was associated with a greater risk of CVD [15]. Thus, visit-to-visit assessment of TyG-related indices may provide more robust CVD risk stratification, as they could represent the long-term effect of TyG-related indices.

To our knowledge, this is the first study to investigate the impact of dynamic TyG-WC, TyG-BMI, and TyG-WHtR levels on CVD events. Our study employed the K-means algorithm to characterize changes in TyG-related indices. Our results revealed that individuals in Class 4, with consistently highest TyG-related indices, had the highest risk of CVD, while those in Class 1, with consistently low TyG-related indices, displayed the lowest CVD risk. Similarly, individuals in Quartile 4 of cumulative TyG-related indices exhibited the highest risk of CVD. Notably, we observed that changes in TyG-WC and cumulative TyG-WC exhibited the strongest association with the risk of CVD. Besides, both changes in TyG-WC and cumulative TyG-WC yielded superior C-statistics in predicting CVD compared with other indices.

The precise mechanism by which TyG-related indices contribute to the development of CVD remains poorly unravelled, with the primary mechanism related to IR warranting consideration. First, IR can trigger a cascade of pathologies, including oxidative stress, chronic inflammatory state, and systemic lipid disturbances, which may lead to the initiation and development of atherosclerosis [41]. Second, IR can induce the overproduction of AGEs, leading to nitric oxide (NO) inactivation, which damages endothelial function [42]. Excessive production of reactive oxygen species (ROS) related to IR also contributes to endothelial dysfunction [43]. Third, IR may mediate platelet activation and adhesion to the endothelium and thrombus formation, leading to arterial stenosis or occlusion, thereby increasing the risk of stroke and heart attack [44, 45]. Moreover, excessive glycosylation triggered by IR can enhance collagen remodelling and smooth muscle cell proliferation, resulting in heightened stiffness and fibrosis of the left ventricle, ultimately raising the susceptibility to heart failure [46]. Last, individuals with IR often accompany a series of validated cardiovascular risk factors, such as a larger WC and BMI, as well as more comorbidities including DM, dyslipidaemia, and hypertension [7, 34]. Notably, our results revealed that the change in TyG-WC showed the strongest association with the incidence of CVD. A cross-sectional study also supported the TyG-WC as a better predictor of total CVD [18]. Our study further corroborated this finding by analysing changes in TyG-WC and cumulative TyG-WC based on repeated assessments. BMI is calculated based on height and weight, which cannot differentiate between fat mass and lean body mass, and therefore cannot accurately reflect the effect of adipose tissue on metabolism [47]. While WC is an indicator of central obesity and may serve as a more accurate predictor for IR. A study supported the point that TyG-WC may be a better predictor for IR [31], partially explaining our finding. Further experimental studies are necessary to validate the specific mechanisms.

Our research enhances the established knowledge by providing evidence that substantiates the utility of dynamic changes in TyG-related indices, particularly TyG-WC, as valuable markers for assessing the risk of CVD. We believe that monitoring long-term TyG-related indices has clinical implications for identifying individuals at high risk of CVD. First, obesity is a recognized risk factor for IR and metabolic disorders. Integrating obesity-related indicators, including WC, BMI, and WHtR, into TyG provides a more comprehensive and robust assessment of IR [31, 48]. Second, our study is based on the CHARLS survey, which regularly conducts regular standardized follow-up on health-related information, offering a reliable data source for analysing dynamic changes in TyG-related indices. Last, height, weight, WC, FBG, and TG are routinely collected and easily available in primary healthcare settings, enhancing the generalizability and practicality of TyG-related indices in clinical implementation.

Nevertheless, we acknowledge certain limitations associated with this study. First, current research cannot directly compare TyG-related indices with the gold standard for IR, which may partially limit the evidence that TyG-related indices, as surrogate markers of IR, increase the risk of CVD events. Second, blood samples in the CHARLS study were only collected only at Wave 1 and Wave 3, limiting our ability to detect more specific dynamic trends in TyG-related indices. Third, since CVD events in the CHARLS were based on questionnaires rather than medical records such as angiography, there may be slight differences in actual CVD incidence. Fourth, in the CHARLS, only one FBG measurement was taken at baseline, and a single measurement of FBG ≥ 126 mg/dL may not be sufficient for diagnosing DM. Fifth, although we adjusted for potential confounders progressively, residual confounder bias cannot be completely ruled out. Last, the individuals in our study were recruited from China and were predominantly middle-aged and older adults; therefore, additional research is necessary to extrapolate these findings to other age groups and countries.

## Conclusion

Our study, based on the CHARLS dataset, indicated that the dynamic changes in TyG-related indices are independently associated with the risk of CVD, with changes in TyG-WC showing the strongest association with CVD incidence. The dynamic changes in TyG-related indices, particularly in TyG-WC, could serve as valuable indicators for CVD risk assessment, thereby providing more information for personalized prevention or intervention strategies for CVD.

### Supplementary Information

Additional file1 (DOCX 434 kb)

## Data Availability

The datasets used in this investigation are available in online repositories. Detailed descriptions of each survey and corresponding data have been published at http://charls.pku.edu.cn/.

## References

[1] Tsao CW, Aday AW, Almarzooq ZI, Anderson CAM, Arora P, Avery CL (2023). Heart disease and stroke statistics—2023 update: a report from the American Heart Association. Circulation.

[2] Roth GA, Mensah GA, Johnson CO, Addolorato G, Ammirati E, Baddour LM (2020). Global burden of cardiovascular diseases and risk factors, 1990–2019: update from the GBD 2019 study. J Am Coll Cardiol.

[3] Vaduganathan M, Mensah GA, Turco JV, Fuster V, Roth GA (2022). The global burden of cardiovascular diseases and risk: a compass for future health. J Am Coll Cardiol.

[4] Wang Z, Ma L, Liu M, Fan J, Hu S, Writing Committee of the Report on Cardiovascular Health and Diseases in China (2023). Summary of the 2022 report on cardiovascular health and diseases in China. Chin Med J (Engl).

[5] Faerch K, Vaag A, Holst JJ, Hansen T, Jørgensen T, Borch-Johnsen K (2009). Natural history of insulin sensitivity and insulin secretion in the progression from normal glucose tolerance to impaired fasting glycemia and impaired glucose tolerance: the Inter99 study. Diabetes Care.

[6] Ormazabal V, Nair S, Elfeky O, Aguayo C, Salomon C, Zuñiga FA (2018). Association between insulin resistance and the development of cardiovascular disease. Cardiovasc Diabetol.

[7] Li M, Chi X, Wang Y, Setrerrahmane S, Xie W, Xu H (2022). Trends in insulin resistance: insights into mechanisms and therapeutic strategy. Signal Transduct Target Ther.

[8] Adeva-Andany MM, Martínez-Rodríguez J, González-Lucán M, Fernández-Fernández C, Castro-Quintela E (2019). Insulin resistance is a cardiovascular risk factor in humans. Diabetes Metab Syndr.

[9] Bornfeldt KE, Tabas I (2011). Insulin resistance, hyperglycemia, and atherosclerosis. Cell Metab.

[10] Otowa-Suematsu N, Sakaguchi K, Kaneko A, Ito J, Morita Y, Miura H (2021). Relation of cardiac function to insulin resistance as evaluated by hyperinsulinemic-euglycemic clamp analysis in individuals with type 2 diabetes. J Diabetes Investig.

[11] Cersosimo E, Solis-Herrera C, Trautmann ME, Malloy J, Triplitt CL (2014). Assessment of pancreatic β-cell function: review of methods and clinical applications. Curr Diabetes Rev.

[12] Mazidi M, Kengne A-P, Katsiki N, Mikhailidis DP, Banach M (2018). Lipid accumulation product and triglycerides/glucose index are useful predictors of insulin resistance. J Diabetes Complicat.

[13] Lee J, Kim B, Kim W, Ahn C, Choi HY, Kim JG (2021). Lipid indices as simple and clinically useful surrogate markers for insulin resistance in the U.S. population. Sci Rep.

[14] Hong S, Han K, Park C-Y (2020). The triglyceride glucose index is a simple and low-cost marker associated with atherosclerotic cardiovascular disease: a population-based study. BMC Med.

[15] Xu X, Huang R, Lin Y, Guo Y, Xiong Z, Zhong X (2022). High triglyceride-glucose index in young adulthood is associated with incident cardiovascular disease and mortality in later life: insight from the CARDIA study. Cardiovasc Diabetol.

[16] Wang A, Tian X, Zuo Y, Chen S, Meng X, Wu S (2021). Change in triglyceride-glucose index predicts the risk of cardiovascular disease in the general population: a prospective cohort study. Cardiovasc Diabetol.

[17] Cui H, Liu Q, Wu Y, Cao L (2022). Cumulative triglyceride-glucose index is a risk for CVD: a prospective cohort study. Cardiovasc Diabetol.

[18] Dang K, Wang X, Hu J, Zhang Y, Cheng L, Qi X (2024). The association between triglyceride-glucose index and its combination with obesity indicators and cardiovascular disease: NHANES 2003–2018. Cardiovasc Diabetol.

[19] Li H, Zuo Y, Qian F, Chen S, Tian X, Wang P (2022). Triglyceride-glucose index variability and incident cardiovascular disease: a prospective cohort study. Cardiovasc Diabetol.

[20] Hao B, Lyu L, Xu J, Zhu X, Xu C, Gao W (2024). The relationship between triglyceride-glucose index and prospective key clinical outcomes in patients hospitalised for coronary artery disease. Cardiovasc Diabetol.

[21] Ahmed B, Sultana R, Greene MW (2021). Adipose tissue and insulin resistance in obese. Biomed Pharmacother.

[22] Liu L, Peng J, Wang N, Wu Z, Zhang Y, Cui H (2024). Comparison of seven surrogate insulin resistance indexes for prediction of incident coronary heart disease risk: a 10-year prospective cohort study. Front Endocrinol (Lausanne).

[23] Park H-M, Han T, Heo S-J, Kwon Y-J (2023). Effectiveness of the triglyceride-glucose index and triglyceride-glucose-related indices in predicting cardiovascular disease in middle-aged and older adults: a prospective cohort study. J Clin Lipidol.

[24] Miao H, Zhou Z, Yang S, Zhang Y (2023). The association of triglyceride-glucose index and related parameters with hypertension and cardiovascular risk: a cross-sectional study. Hypertens Res.

[25] Huo R-R, Zhai L, Liao Q, You X-M (2023). Changes in the triglyceride glucose-body mass index estimate the risk of stroke in middle-aged and older Chinese adults: a nationwide prospective cohort study. Cardiovasc Diabetol.

[26] Li F, Wang Y, Shi B, Sun S, Wang S, Pang S (2024). Association between the cumulative average triglyceride glucose-body mass index and cardiovascular disease incidence among the middle-aged and older population: a prospective nationwide cohort study in China. Cardiovasc Diabetol.

[27] Zhao Y, Hu Y, Smith JP, Strauss J, Yang G (2014). Cohort profile: the China health and retirement longitudinal study (CHARLS). Int J Epidemiol.

[28] Sinaga KP, Yang M-S (2020). Unsupervised K-means clustering algorithm. IEEE Access.

[29] Singh A, Yadav A, Rana A (2013). K-means with three different distance metrics. IJCA.

[30] Ma Y-C, Zuo L, Chen J-H, Luo Q, Yu X-Q, Li Y (2006). Modified glomerular filtration rate estimating equation for Chinese patients with chronic kidney disease. J Am Soc Nephrol.

[31] Yan S, Wang D, Jia Y (2023). Comparison of insulin resistance-associated parameters in US adults: a cross-sectional study. Hormones (Athens).

[32] Tuo X, Yuan J, Wang X-H, Xin Z (2020). Identifying the insulin resistance index in nondiabetic Chinese subjects. Medicine (Baltimore).

[33] Ruiz HH, Ramasamy R, Schmidt AM (2019). Advanced glycation end products: building on the concept of the “common soil” in metabolic disease. Endocrinology.

[34] Shulman GI (2014). Ectopic fat in insulin resistance, dyslipidemia, and cardiometabolic disease. N Engl J Med.

[35] Pinto-Junior DC, Silva KS, Michalani ML, Yonamine CY, Esteves JV, Fabre NT (2018). Advanced glycation end products-induced insulin resistance involves repression of skeletal muscle GLUT4 expression. Sci Rep.

[36] Tan KCB, Shiu SWM, Wong Y, Tam X (2011). Serum advanced glycation end products (AGEs) are associated with insulin resistance. Diabetes Metab Res Rev.

[37] Matsuda M, Shimomura I (2013). Increased oxidative stress in obesity: implications for metabolic syndrome, diabetes, hypertension, dyslipidemia, atherosclerosis, and cancer. Obes Res Clin Pract.

[38] Shao Y, Hu H, Li Q, Cao C, Liu D, Han Y (2024). Link between triglyceride-glucose-body mass index and future stroke risk in middle-aged and elderly Chinese: a nationwide prospective cohort study. Cardiovasc Diabetol.

[39] Wang Z, He H, Xie Y, Li J, Luo F, Sun Z (2024). Non-insulin-based insulin resistance indexes in predicting atrial fibrillation recurrence following ablation: a retrospective study. Cardiovasc Diabetol.

[40] Liu Z, Deng B, Huang Q, Tu R, Yu F, Xia J (2023). Comparison of seven surrogate insulin resistance indexes for predicting the prevalence of carotid atherosclerosis in normal-weight individuals. Front Public Health.

[41] Yang Q, Vijayakumar A, Kahn BB (2018). Metabolites as regulators of insulin sensitivity and metabolism. Nat Rev Mol Cell Biol.

[42] Molina MN, Ferder L, Manucha W (2016). Emerging role of nitric oxide and heat shock proteins in insulin resistance. Curr Hypertens Rep.

[43] Nishikawa T, Araki E (2007). Impact of mitochondrial ROS production in the pathogenesis of diabetes mellitus and its complications. Antioxid Redox Signal.

[44] Kaur R, Kaur M, Singh J (2018). Endothelial dysfunction and platelet hyperactivity in type 2 diabetes mellitus: molecular insights and therapeutic strategies. Cardiovasc Diabetol.

[45] Tao L-C, Xu J-N, Wang T-T, Hua F, Li J-J (2022). Triglyceride-glucose index as a marker in cardiovascular diseases: landscape and limitations. Cardiovasc Diabetol.

[46] Hill MA, Yang Y, Zhang L, Sun Z, Jia G, Parrish AR (2021). Insulin resistance, cardiovascular stiffening and cardiovascular disease. Metabolism.

[47] Lee DH, Keum N, Hu FB, Orav EJ, Rimm EB, Willett WC (2018). Predicted lean body mass, fat mass, and all cause and cause specific mortality in men: prospective US cohort study. BMJ.

[48] Lim J, Kim J, Koo SH, Kwon GC (2019). Comparison of triglyceride glucose index, and related parameters to predict insulin resistance in Korean adults: an analysis of the 2007–2010 Korean national health and nutrition examination survey. PLoS ONE.

