# Supplementary material for: Changes in the combination of the triglyceride-glucose index and obesity indicators estimate the risk of cardiovascular disease
Source: Cardiovasc Diabetol. 2024 Jun 6;23:192. doi: 10.1186/s12933-024-02281-4 (PMC11157789; doi:10.1186/s12933-024-02281-4)
Supplement: Supplementary file 1 — Additional file1 (DOCX 434 kb) [file 12933_2024_2281_MOESM1_ESM.docx]

**Changes in the combination of the triglyceride-glucose index and obesity indicators estimate the risk of cardiovascular disease**

**Contents:**

1. Table S1 Baseline characteristics of 3243 participants according to TyG-BMI change classes.
2. Table S2 Baseline characteristics of 3243 participants according to TyG-WHtR change classes.
3. Table S3 Associations of changes in TyG-BMI and cumulative TyG-BMI with cardiovascular disease incidence.
4. Table S4 Associations of changes in TyG-WHtR and cumulative TyG-WHtR with cardiovascular disease incidence.
5. Table S5 Associations of changes in TyG and cumulative TyG with cardiovascular disease incidence.
6. Table S6 Incremental value of changes in TyG-WC for incident CVD.
7. Table S7 Incremental value of changes in TyG-BMI for incident CVD.
8. Table S8 Incremental value of changes in TyG-WHtR for incident CVD.
9. Table S9 Associations of changes in TyG-WC and cumulative TyG-WC with cardiovascular disease incidence in complete dataset.
10. Table S10 Associations of changes in TyG-BMI and cumulative TyG-BMI with cardiovascular disease incidence in complete dataset.
11. Table S11 Associations of changes in TyG-WHtR and cumulative TyG-WHtR with cardiovascular disease incidence in complete dataset.
12. Fig. S1 Clustering of changes in TyG-BMI from Wave 1 to Wave 3.
13. Fig. S2 Clustering of changes in TyG-WHtR from Wave 1 to Wave 3.

**Table S1** Baseline characteristics of 3243 participants according to TyG-BMI change classes

| **Characteristic** | **Overall (n = 3243)** | **Changes in TyG-BMI** | | | | |
| --- | --- | --- | --- | --- | --- | --- |
|  |  | **Class 1 (n = 940)** | **Class 2 (n = 1211)** | **Class 3 (n = 781)** | **Class 4 (n = 311)** | ***P value*** |
| Age, mean ± SD, years | 57.62 ± 8.40 | 59.58 ± 8.86 | 57.49 ± 8.32 | 56.27 ± 7.90 | 55.66 ± 7.25 | < 0.001 |
| Sex |  |  |  |  |  | < 0.001 |
| Female | 1761 (54.4) | 389 (41.5) | 674 (55.7) | 487 (62.4) | 211 (68.1) |  |
| Male | 1477 (45.6) | 548 (58.5) | 536 (44.3) | 294 (37.6) | 99 (31.9) |  |
| Marital status, n (%) |  |  |  |  |  | 0.003 |
| Married | 2810 (86.6) | 792 (84.3) | 1041 (86) | 693 (88.7) | 284 (91.3) |  |
| Others | 433 (13.4) | 148 (15.7) | 170 (14) | 88 (11.3) | 27 (8.7) |  |
| Education level, n (%) |  |  |  |  |  | 0.052 |
| No formal education | 927 (28.6) | 274 (29.1) | 357 (29.5) | 204 (26.1) | 92 (29.6) |  |
| Primary school | 1340 (41.3) | 418 (44.5) | 485 (40) | 314 (40.2) | 123 (39.5) |  |
| Middle or high  school | 896 (27.6) | 234 (24.9) | 332 (27.4) | 241 (30.9) | 89 (28.6) |  |
| College or above | 80 (2.5) | 14 (1.5) | 37 (3.1) | 22 (2.8) | 7 (2.3) |  |
| Smoking status^a^, n (%) |  |  |  |  |  | < 0.001 |
| Never | 2026 (62.6) | 465 (49.7) | 780 (64.5) | 553 (71) | 228 (73.3) |  |
| Former | 225 (7.0) | 63 (6.7) | 76 (6.3) | 66 (8.5) | 20 (6.4) |  |
| Current | 983 (30.4) | 407 (43.5) | 353 (29.2) | 160 (20.5) | 63 (20.3) |  |
| Drinking status^a^, n (%) |  |  |  |  |  | < 0.001 |
| Never | 1890 (58.4) | 483 (51.5) | 716 (59.2) | 476 (61.1) | 215 (69.1) |  |
| Former | 230 (7.1) | 76 (8.1) | 73 (6) | 63 (8.1) | 18 (5.8) |  |
| Current | 1117 (34.5) | 378 (40.3) | 421 (34.8) | 240 (30.8) | 78 (25.1) |  |
| Hypertension^a^, n (%) |  |  |  |  |  | < 0.001 |
| No | 2066 (64.1) | 715 (76.6) | 816 (67.7) | 412 (53) | 123 (39.7) |  |
| Yes | 1159 (35.9) | 218 (23.4) | 389 (32.3) | 365 (47) | 187 (60.3) |  |
| Diabetes^a^, n (%) |  |  |  |  |  | < 0.001 |
| No | 2801 (87.1) | 871 (93.3) | 1088 (90.4) | 626 (81.1) | 216 (70.1) |  |
| Yes | 416 (12.9) | 63 (6.7) | 115 (9.6) | 146 (18.9) | 92 (29.9) |  |
| Dyslipidaemia^a^, n (%) |  |  |  |  |  | < 0.001 |
| No | 1694 (52.9) | 711 (76.7) | 674 (56.5) | 256 (33) | 53 (17.3) |  |
| Yes | 1507 (47.1) | 216 (23.3) | 518 (43.5) | 520 (67) | 253 (82.7) |  |
| Kidney disease^a^, n (%) |  |  |  |  |  | 0.123 |
| No | 3048 (94.8) | 877 (93.9) | 1137 (94.8) | 734 (94.8) | 300 (97.4) |  |
| Yes | 167 (5.2) | 57 (6.1) | 62 (5.2) | 40 (5.2) | 8 (2.6) |  |
| Systolic Blood pressure,  mean ± SD, mmHg^a^ | 128.58 ± 20.37 | 123.83 ± 19.67 | 127.50 ± 19.52 | 132.50 ± 20.34 | 137.45 ± 21.16 | < 0.001 |
| Diastolic Blood pressure,  mean ± SD, mmHg^a^ | 75.29 ± 11.77 | 71.68 ± 11.00 | 74.80 ± 11.39 | 77.98 ± 11.61 | 81.38 ± 11.92 | < 0.001 |
| TC, mean ± SD, mg/dl | 194.12 ± 38.74 | 185.35 ± 36.25 | 193.47 ± 36.72 | 200.91 ± 37.75 | 206.10 ± 48.71 | < 0.001 |
| HDL-C, mean ± SD, mg/dl | 51.30 ± 15.15 | 59.09 ± 16.22 | 52.08 ± 13.45 | 45.12 ± 11.91 | 40.25 ± 11.59 | < 0.001 |
| LDL-C, mean ± SD, mg/dl^a^ | 117.15 ± 34.55 | 110.78 ± 31.32 | 119.75 ± 33.45 | 122.09 ± 36.68 | 113.85 ± 39.29 | < 0.001 |
| HbA1c, mean ± SD, % | 5.27 ± 0.82 | 5.12 ± 0.58 | 5.19 ± 0.68 | 5.43 ± 1.02 | 5.65 ± 1.14 | < 0.001 |
| eGFR, mean ± SD, ml/min/1.73 m^2a^ | 110.77 ± 29.11 | 110.88 ± 27.81 | 110.53 ± 27.59 | 110.56 ± 29.93 | 111.84 ± 35.95 | 0.906 |
| FBG_2012_, mean ± SD, mg/dl | 109.08 ± 33.94 | 102.16 ± 22.80 | 105.20 ± 24.91 | 115.93 ± 42.18 | 127.92 ± 53.82 | < 0.001 |
| TG_2012_, median (IQR), mg/dl | 101.78 (72.57, 147.79) | 74.34 (58.41, 100.00) | 97.35 (74.34, 134.08) | 138.06 (100.89, 192.04) | 185.85 (130.54, 280.99) | < 0.001 |
| hsCRP_2012_, median (IQR), mg/L | 0.98 (0.53, 1.97) | 0.76 (0.44, 1.62) | 0.88 (0.50, 1.77) | 1.21 (0.69, 2.22) | 1.62 (0.91, 2.85) | < 0.001 |
| TyG_2012_, mean ± SD | 8.66 ± 0.66 | 8.26 ± 0.47 | 8.56 ± 0.50 | 8.98 ± 0.61 | 9.39 ± 0.79 | < 0.001 |
| TyG_2015_, mean ± SD | 8.66 ± 0.61 | 8.23 ± 0.41 | 8.58 ± 0.47 | 9.01 ± 0.55 | 9.39 ± 0.61 | < 0.001 |
| BMI_2012_, mean ± SD, kg/m² | 23.51 ± 3.70 | 19.88 ± 1.70 | 23.07 ± 1.59 | 25.99 ± 1.93 | 30.01 ± 4.10 | < 0.001 |
| BMI_2015_, mean ± SD, kg/m² | 23.69 ± 3.55 | 19.92 ± 1.70 | 23.43 ± 1.61 | 26.26 ± 2.04 | 29.61 ± 2.76 | < 0.001 |
| WC_2012_, mean ± SD, cm | 85.13 ± 9.64 | 76.32 ± 6.08 | 84.13 ± 6.25 | 91.65 ± 6.65 | 99.26 ± 7.11 | < 0.001 |
| WC_2015_, mean ± SD, cm | 85.90 ± 10.06 | 76.21 ± 6.34 | 85.44 ± 6.21 | 92.45 ± 6.95 | 100.50 ± 7.58 | < 0.001 |
| WHtR_2012_, mean ± SD | 0.54 ± 0.06 | 0.48 ± 0.04 | 0.53 ± 0.04 | 0.58 ± 0.05 | 0.63 ± 0.05 | < 0.001 |
| WHtR_2015_, mean ± SD | 0.55 ± 0.07 | 0.48 ± 0.04 | 0.54 ± 0.04 | 0.59 ± 0.05 | 0.64 ± 0.05 | < 0.001 |
| TyG-BMI_2012_, mean ± SD | 204.18 ± 39.55 | 164.06 ± 14.88 | 197.18 ± 14.05 | 232.88 ± 17.09 | 280.65 ± 34.13 | < 0.001 |
| TyG-BMI_2015_, mean ± SD | 205.96 ± 38.66 | 163.93 ± 14.80 | 200.87 ± 14.06 | 236.00 ± 16.59 | 277.40 ± 25.57 | < 0.001 |
| TyG-WC_2012_, mean ± SD | 738.92 ± 116.04 | 630.38 ± 59.36 | 719.90 ± 64.32 | 822.47 ± 74.15 | 931.26 ± 91.26 | < 0.001 |
| TyG-WC_2015_, mean ± SD | 746.43 ± 118.41 | 627.56 ± 60.63 | 733.07 ± 62.23 | 832.12 ± 71.59 | 942.57 ± 82.78 | < 0.001 |
| TyG-WHtR_2012_,  mean ± SD | 4.69 ± 0.75 | 3.98 ± 0.39 | 4.57 ± 0.43 | 5.22 ± 0.50 | 5.91 ± 0.57 | < 0.001 |
| TyG-WHtR_2015_,  mean ± SD | 4.75 ± 0.78 | 3.98 ± 0.41 | 4.67 ± 0.44 | 5.30 ± 0.49 | 5.99 ± 0.52 | < 0.001 |
| Cumulative TyG-BMI,  mean ± SD | 615.2 ± 111.8 | 492.0 ± 37.6 | 597.1 ± 30.0 | 703.3 ± 33.3 | 837.1 ± 62.7 | < 0.001 |
| Cumulative TyG-WC,  mean ± SD | 2228.0 ± 332.6 | 1886.9 ± 151.8 | 2179.5 ± 155.4 | 2481.9 ± 177.9 | 2810.7 ± 219.3 | < 0.001 |
| Cumulative TyG-WHtR, mean ± SD | 14.15 ± 2.17 | 11.94 ± 1.04 | 13.86 ± 1.10 | 15.78 ± 1.24 | 17.86 ± 1.36 | < 0.001 |

BMI: body mass index; WC: waist circumference; WHtR: waist height ratio; eGFR: estimated glomerular filtration ratio; HbA1c: glycated haemoglobin; FBG: fasting plasma glucose; TG: triglyceride; hsCRP: hypersensitive C-reactive protein; HDL-C: high-density lipoprotein cholesterol; LDL-C: low-density lipoprotein cholesterol; SD: standard deviation; TC: total cholesterol; TyG: triglyceride-glucose index

^a^ Missing data: 5 for sex, 9 for smoking status, 6 for drinking status, 18 for hypertension, 26 for diabetes, 42 for dyslipidaemia, 28 for kidney disease, 13 for systolic blood pressure, 13 for diastolic blood pressure, 7 for LDL-C, 1 for the eGFR, and 11 for HbA1c.

**Table S2** Baseline characteristics of 3243 participants according to TyG-WHtR change classes

| **Characteristic** | | **Overall (n = 3243)** | **Changes in TyG-WHtR** | | | | |
| --- | --- | --- | --- | --- | --- | --- | --- |
|  | |  | **Class 1 (n = 919)** | **Class 2 (n = 1110)** | **Class 3 (n = 814)** | **Class 4 (n = 400)** | ***P value*** |
| Age, mean ± SD, years | | 57.62 ± 8.40 | 57.88 ± 8.53 | 57.25 ± 8.34 | 57.70 ± 8.47 | 57.94 ± 8.11 | 0.294 |
| Sex | |  |  |  |  |  | < 0.001 |
| Female | | 1761 (54.4) | 292 (31.8) | 616 (55.6) | 545 (67) | 308 (77.2) |  |
| Male, n (%) | | 1477 (45.6) | 626 (68.2) | 491 (44.4) | 269 (33) | 91 (22.8) |  |
| Marital status | |  |  |  |  |  | 0.981 |
| Married | | 2810 (86.6) | 797 (86.7) | 962 (86.7) | 707 (86.9) | 344 (86) |  |
| Others | | 433 (13.4) | 122 (13.3) | 148 (13.3) | 107 (13.1) | 56 (14) |  |
| Education level, n (%) | |  |  |  |  |  | < 0.001 |
| No formal education | | 927 (28.6) | 216 (23.5) | 305 (27.5) | 268 (32.9) | 138 (34.5) |  |
| Primary school | | 1340 (41.3) | 403 (43.9) | 461 (41.5) | 311 (38.2) | 165 (41.2) |  |
| Middle or high  school | | 896 (27.6) | 279 (30.4) | 312 (28.1) | 216 (26.5) | 89 (22.2) |  |
| College or above | | 80 (2.5) | 21 (2.3) | 32 (2.9) | 19 (2.3) | 8 (2) |  |
| Smoking status^a^, n (%) | |  |  |  |  |  | < 0.001 |
| Never | | 2026 (62.6) | 399 (43.6) | 718 (64.7) | 587 (72.4) | 322 (80.7) |  |
| Former | | 225 (7.0) | 73 (8) | 78 (7) | 52 (6.4) | 22 (5.5) |  |
| Current | | 983 (30.4) | 443 (48.4) | 313 (28.2) | 172 (21.2) | 55 (13.8) |  |
| Drinking status^a^, n (%) | |  |  |  |  |  | < 0.001 |
| Never | | 1890 (58.4) | 423 (46.2) | 661 (59.6) | 518 (63.8) | 288 (72) |  |
| Former | | 230 (7.1) | 73 (8) | 73 (6.6) | 55 (6.8) | 29 (7.2) |  |
| Current | | 1117 (34.5) | 420 (45.9) | 375 (33.8) | 239 (29.4) | 83 (20.8) |  |
| Hypertension^a^, n (%) | |  |  |  |  |  | < 0.001 |
| No | | 2066 (64.1) | 720 (79) | 757 (68.6) | 432 (53.3) | 157 (39.3) |  |
| Yes | | 1159 (35.9) | 191 (21) | 347 (31.4) | 379 (46.7) | 242 (60.7) |  |
| Diabetes^a^, n (%) | |  |  |  |  |  | < 0.001 |
| No | | 2801 (87.1) | 848 (92.9) | 1010 (91.7) | 685 (85) | 258 (65.2) |  |
| Yes | | 416 (12.9) | 65 (7.1) | 92 (8.3) | 121 (15) | 138 (34.8) |  |
| Dyslipidaemia^a^, n (%) | |  |  |  |  |  | < 0.001 |
| No | | 1694 (52.9) | 697 (77) | 646 (59.1) | 292 (36.1) | 59 (14.9) |  |
| Yes | | 1507 (47.1) | 208 (23) | 447 (40.9) | 516 (63.9) | 336 (85.1) |  |
| Kidney disease^a^, n (%) | |  |  |  |  |  | 0.258 |
| No | | 3048 (94.8) | 855 (94.1) | 1045 (94.7) | 765 (94.9) | 383 (96.7) |  |
| Yes | | 167 (5.2) | 54 (5.9) | 59 (5.3) | 41 (5.1) | 13 (3.3) |  |
| Systolic Blood pressure,  mean ± SD, mmHg^a^ | | 128.58 ± 20.37 | 122.44 ± 17.96 | 127.22 ± 19.48 | 132.74 ± 20.72 | 138.12 ± 21.91 | < 0.001 |
| Diastolic Blood pressure,  mean ± SD, mmHg^a^ | | 75.29 ± 11.77 | 71.73 ± 11.12 | 74.72 ± 11.09 | 77.64 ± 11.98 | 80.26 ± 11.91 | < 0.001 |
| TC, mean ± SD, mg/dl | | 194.12 ± 38.74 | 182.94 ± 35.71 | 192.54 ± 35.36 | 201.26 ± 37.45 | 209.67 ± 47.82 | < 0.001 |
| HDL-C, mean ± SD, mg/dl | | 51.30 ± 15.15 | 59.01 ± 16.32 | 52.27 ± 13.35 | 46.64 ± 12.48 | 40.40 ± 11.65 | < 0.001 |
| LDL-C, mean ± SD, mg/dl^a^ | | 117.15 ± 34.55 | 109.28 ± 31.17 | 119.73 ± 31.63 | 123.11 ± 36.01 | 115.94 ± 42.61 | < 0.001 |
| HbA1c, mean ± SD, % | | 5.27 ± 0.82 | 5.11 ± 0.57 | 5.18 ± 0.65 | 5.33 ± 0.85 | 5.78 ± 1.31 | < 0.001 |
| eGFR, mean ± SD, ml/min/1.73 m^2a^ | | 110.77 ± 29.11 | 110.96 ± 25.21 | 110.47 ± 28.74 | 110.04 ± 31.03 | 112.60 ± 34.05 | 0.522 |
| FBG_2012_, mean ± SD, mg/dl | 109.08 ± 33.94 | 101.41 ± 19.42 | 104.86 ± 26.99 | 111.10 ± 30.00 | 134.31 ± 61.67 | < 0.001 | |
| TG_2012_, median (IQR), mg/dl | 101.78 (72.57, 147.79) | 71.68 (56.64, 93.81) | 97.35 (74.34, 130.98) | 131.87 (98.46, 182.09) | 191.60 (138.06, 287.62) | < 0.001 | |
| hsCRP_2012_, median (IQR), mg/L | 0.98 (0.53, 1.97) | 0.74 (0.43, 1.49) | 0.84 (0.49, 1.78) | 1.20 (0.69, 2.23) | 1.54 (0.90, 2.94) | < 0.001 | |
| TyG_2012_, mean ± SD | | 8.66 ± 0.66 | 8.22 ± 0.43 | 8.54 ± 0.47 | 8.91 ± 0.56 | 9.47 ± 0.76 | < 0.001 |
| TyG_2015_, mean ± SD | | 8.66 ± 0.61 | 8.20 ± 0.38 | 8.57 ± 0.44 | 8.94 ± 0.53 | 9.41 ± 0.59 | < 0.001 |
| BMI_2012_, mean ± SD, kg/m² | | 23.51 ± 3.70 | 20.38 ± 2.13 | 23.07 ± 2.79 | 25.38 ± 2.83 | 28.14 ± 3.37 | < 0.001 |
| BMI_2015_, mean ± SD, kg/m² | | 23.69 ± 3.55 | 20.49 ± 2.22 | 23.30 ± 2.32 | 25.60 ± 2.58 | 28.23 ± 3.27 | < 0.001 |
| WC_2012_, mean ± SD, cm | | 85.13 ± 9.64 | 76.23 ± 6.00 | 83.61 ± 6.39 | 90.81 ± 6.42 | 98.22 ± 7.00 | < 0.001 |
| WC_2015_, mean ± SD, cm | | 85.90 ± 10.06 | 76.06 ± 6.46 | 84.93 ± 6.22 | 91.98 ± 6.72 | 98.80 ± 7.73 | < 0.001 |
| WHtR_2012_, mean ± SD | | 0.54 ± 0.06 | 0.47 ± 0.03 | 0.53 ± 0.03 | 0.58 ± 0.04 | 0.64 ± 0.04 | < 0.001 |
| WHtR_2015_, mean ± SD | | 0.55 ± 0.07 | 0.47 ± 0.04 | 0.54 ± 0.03 | 0.59 ± 0.04 | 0.64 ± 0.05 | < 0.001 |
| TyG-BMI_2012_, mean ± SD | | 204.18 ± 39.55 | 167.37 ± 18.54 | 196.70 ± 23.89 | 225.79 ± 25.73 | 265.56 ± 31.23 | < 0.001 |
| TyG-BMI_2015_, mean ± SD | | 205.96 ± 38.66 | 167.88 ± 19.37 | 199.54 ± 20.58 | 228.62 ± 23.79 | 265.20 ± 30.50 | < 0.001 |
| TyG-WC_2012_, mean ± SD | | 738.92 ± 116.04 | 626.24 ± 57.14 | 713.30 ± 59.61 | 808.04 ± 64.72 | 928.27 ± 82.02 | < 0.001 |
| TyG-WC_2015_, mean ± SD | | 746.43 ± 118.41 | 623.29 ± 58.37 | 727.41 ± 58.89 | 821.73 ± 67.12 | 928.90 ± 79.95 | < 0.001 |
| TyG-WHtR_2012_,  mean ± SD | | 4.69 ± 0.75 | 3.89 ± 0.31 | 4.52 ± 0.29 | 5.16 ± 0.32 | 6.00 ± 0.45 | < 0.001 |
| TyG-WHtR_2015_,  mean ± SD | | 4.75 ± 0.78 | 3.88 ± 0.32 | 4.62 ± 0.31 | 5.27 ± 0.34 | 6.02 ± 0.46 | < 0.001 |
| Cumulative TyG-BMI,  mean ± SD | | 615.2 ± 111.8 | 502.9 ± 50.7 | 594.3 ± 56.5 | 681.6 ± 63.1 | 796.1 ± 81.4 | < 0.001 |
| Cumulative TyG-WC,  mean ± SD | | 2228.0 ± 332.6 | 1874.3 ± 141.2 | 2161.1 ± 138.9 | 2444.7 ± 157.8 | 2785.8 ± 200.9 | < 0.001 |
| Cumulative TyG-WHtR, mean ± SD | | 14.15 ± 2.17 | 11.67 ± 0.72 | 13.71 ± 0.56 | 15.64 ± 0.63 | 18.02 ± 1.03 | < 0.001 |

BMI: body mass index; WC: waist circumference; WHtR: waist height ratio; eGFR: estimated glomerular filtration ratio; HbA1c: glycated haemoglobin; FBG: fasting plasma glucose; TG: triglyceride; hsCRP: hypersensitive C-reactive protein; HDL-C: high-density lipoprotein cholesterol; LDL-C: low-density lipoprotein cholesterol; SD: standard deviation; TC: total cholesterol; TyG: triglyceride-glucose index

^a^ Missing data: 5 for sex, 9 for smoking status, 6 for drinking status, 18 for hypertension, 26 for diabetes, 42 for dyslipidaemia, 28 for kidney disease, 13 for systolic blood pressure, 13 for diastolic blood pressure, 7 for LDL-C, 1 for the eGFR, and 11 for HbA1c

**Table S3** Associations of changes in TyG-BMI and cumulative TyG-BMI with cardiovascular disease incidence

|  | **Event/total** | **Model 1^a^** |  | **Model 2^b^** |  | **Model 3^c^** |  | **Model 4^d^** |  |
| --- | --- | --- | --- | --- | --- | --- | --- | --- | --- |
|  |  | **OR (95%CI)** | **P value** | **OR (95%CI)** | **P value** | **OR (95%CI)** | **P value** | **OR (95%CI)** | **P value** |
| **Changes in TyG-BM**I |  |  |  |  |  |  |  |  |  |
| Class1 | 139/940 | Reference |  | Reference |  | Reference |  | Reference |  |
| Class2 | 241/1211 | 1.52 (1.2~1.92) | <0.001 | 1.52 (1.2~1.92) | <0.001 | 1.34 (1.06~1.71) | 0.016 | 1.32 (1.03~1.68) | 0.029 |
| Class3 | 167/781 | 1.72 (1.33~2.23) | <0.001 | 1.71 (1.32~2.22) | <0.001 | 1.28 (0.96~1.69) | 0.092 | 1.22 (0.91~1.64) | 0.185 |
| Class4 | 90/311 | 2.63 (1.93~3.61) | <0.001 | 2.63 (1.92~3.6) | <0.001 | 1.69 (1.18~2.41) | 0.004 | 1.68 (1.15~2.46) | 0.007 |
| **Cumulative TyG-BMI** |  |  |  |  |  |  |  |  |  |
| Quartile1 | 118/811 | Reference |  | Reference |  | Reference |  | Reference |  |
| Quartile2 | 154/810 | 1.45 (1.12~1.90) | 0.006 | 1.45 (1.11~1.89) | 0.007 | 1.32 (1.01~1.73) | 0.044 | 1.28 (0.98~1.69) | 0.074 |
| Quartile3 | 158/811 | 1.57 (1.20~2.05) | 0.001 | 1.56 (1.19~2.04) | 0.001 | 1.29 (0.98~1.72) | 0.073 | 1.26 (0.94~1.68) | 0.126 |
| Quartile4 | 207/811 | 2.26 (1.74~2.93) | <0.001 | 2.26 (1.73~2.94) | <0.001 | 1.55 (1.15~2.1) | 0.004 | 1.49 (1.09~2.05) | 0.014 |
| P for trend | 637/3243 | 1.29 (1.19~1.40) | <0.001 | 1.29 (1.18~1.4) | <0.001 | 1.14 (1.03~1.25) | 0.009 | 1.12 (1.01~1.24) | 0.026 |

BMI: body mass index; CI: confidence interval; OR: odds ratio; TyG: triglyceride-glucose index

a Adjusted for age and sex

b Adjusted for age, sex, marital status, educational level, smoking status, and drinking status

c Adjusted for variables in Model 2 and history of hypertension, diabetes, dyslipidaemia, kidney disease, medication use for hypertension, medication use for diabetes, medication use for dyslipidaemia, systolic blood pressure, diastolic blood pressure

d Adjusted for variables in Model 3 and total cholesterol, HDL-C, LDL-C, HbA1c, the eGFR, and hsCRP

**Table S4** Associations of changes in TyG-WHtR and cumulative TyG-WHtR with cardiovascular disease incidence

|  | **Event/total** | **Model 1^a^** |  | **Model 2^b^** |  | **Model 3^c^** |  | **Model 4^d^** |  |
| --- | --- | --- | --- | --- | --- | --- | --- | --- | --- |
|  |  | **OR (95%CI)** | **P value** | **OR (95%CI)** | **P value** | **OR (95%CI)** | **P value** | **OR (95%CI)** | **P value** |
| **Changes in TyG-WHtR** |  |  |  |  |  |  |  |  |  |
| Class1 | 130/919 | Reference |  | Reference |  | Reference |  | Reference |  |
| Class2 | 211/1110 | 1.44 (1.13~1.84) | 0.003 | 1.43 (1.12~1.83) | 0.004 | 1.27 (0.99~1.64) | 0.06 | 1.23 (0.95~1.59) | 0.116 |
| Class3 | 181/814 | 1.73 (1.33~2.24) | <0.001 | 1.72 (1.33~2.23) | <0.001 | 1.31 (0.99~1.74) | 0.061 | 1.25 (0.93~1.68) | 0.141 |
| Class4 | 115/400 | 2.42 (1.79~3.27) | <0.001 | 2.39 (1.77~3.24) | <0.001 | 1.57 (1.10~2.22) | 0.012 | 1.53 (1.05~2.24) | 0.026 |
| **Cumulative TyG-WHtR** |  |  |  |  |  |  |  |  |  |
| Quartile1 | 111/811 | Reference |  | Reference |  | Reference |  | Reference |  |
| Quartile2 | 146/810 | 1.41 (1.08~1.85) | 0.013 | 1.41 (1.07~1.85) | 0.014 | 1.26 (0.96~1.67) | 0.098 | 1.23 (0.93~1.63) | 0.150 |
| Quartile3 | 168/811 | 1.67 (1.27~2.19) | <0.001 | 1.66 (1.27~2.18) | <0.001 | 1.39 (1.04~1.84) | 0.026 | 1.32 (0.98~1.78) | 0.065 |
| Quartile4 | 212/811 | 2.23 (1.7~2.92) | <0.001 | 2.23 (1.7~2.92) | <0.001 | 1.52 (1.12~2.08) | 0.008 | 1.47 (1.05~2.04) | 0.024 |
| P for trend | 637/3243 | 1.29 (1.19~1.4) | <0.001 | 1.29 (1.19~1.4) | <0.001 | 1.14 (1.03~1.26) | 0.009 | 1.13 (1.01~1.25) | 0.026 |

WHtR: waist height ratio; CI: confidence interval; OR: odds ratio; TyG: triglyceride-glucose index

a Adjusted for age and sex

b Adjusted for age, sex, marital status, educational level, smoking status, and drinking status

c Adjusted for variables in Model 2 and history of hypertension, diabetes, dyslipidaemia, kidney disease, medication use for hypertension, medication use for diabetes, medication use for dyslipidaemia, systolic blood pressure, diastolic blood pressure

d Adjusted for variables in Model 3 and total cholesterol, HDL-C, LDL-C, HbA1c, the eGFR, and hsCRP

**Table S5** Associations of changes in TyG and cumulative TyG with cardiovascular disease incidence

|  | **Event/total** | **Model 1^a^** |  | **Model 2^b^** |  | **Model 3^c^** |  | **Model 4^d^** |  |
| --- | --- | --- | --- | --- | --- | --- | --- | --- | --- |
|  |  | **OR (95%CI)** | **P value** | **OR (95%CI)** | **P value** | **OR (95%CI)** | **P value** | **OR (95%CI)** | **P value** |
| **Changes in TyG** |  |  |  |  |  |  |  |  |  |
| Class1 | 152/1009 | Reference |  | Reference |  | Reference |  | Reference |  |
| Class2 | 232/1174 | 1.37 (1.09~1.72) | 0.006 | 1.38 (1.10~1.73) | 0.006 | 1.25 (0.99~1.59) | 0.061 | 1.17 (0.92~1.50) | 0.197 |
| Class3 | 178/766 | 1.68 (1.32~2.15) | <0.001 | 1.67 (1.31~2.13) | <0.001 | 1.31 (0.99~1.72) | 0.058 | 1.19 (0.89~1.60) | 0.243 |
| Class4 | 75/294 | 1.94 (1.41~2.66) | <0.001 | 1.93 (1.40~2.65) | <0.001 | 1.27 (0.85~1.88) | 0.240 | 1.19 (0.73~1.95) | 0.482 |
| **Cumulative TyG** |  |  |  |  |  |  |  |  |  |
| Quartile1 | 119/811 | Reference |  | Reference |  | Reference |  | Reference |  |
| Quartile2 | 153/810 | 1.34 (1.03~1.75) | 0.029 | 1.34 (1.03~1.75) | 0.029 | 1.23 (0.94~1.62) | 0.130 | 1.18 (0.89~1.55) | 0.247 |
| Quartile3 | 171/811 | 1.55 (1.19~2.01) | 0.001 | 1.54 (1.19~2.00) | 0.001 | 1.33 (1.01~1.75) | 0.046 | 1.20 (0.90~1.60) | 0.212 |
| Quartile4 | 194/811 | 1.82 (1.41~2.35) | <0.001 | 1.80 (1.39~2.33) | <0.001 | 1.25 (0.91~1.71) | 0.165 | 1.12 (0.79~1.59) | 0.516 |
| P for trend | 637/3243 | 1.21 (1.12~1.31) | <0.001 | 1.21 (1.11~1.31) | <0.001 | 1.08 (0.98~1.19) | 0.139 | 1.04 (0.93~1.16) | 0.466 |

CI: confidence interval; OR: odds ratio; TyG: triglyceride-glucose index

a Adjusted for age and sex

b Adjusted for age, sex, marital status, educational level, smoking status, and drinking status

c Adjusted for variables in Model 2 and history of hypertension, diabetes, dyslipidaemia, kidney disease, medication use for hypertension, medication use for diabetes, medication use for dyslipidaemia, systolic blood pressure, diastolic blood pressure

d Adjusted for variables in Model 3 and total cholesterol, HDL-C, LDL-C, HbA1c, the eGFR, and hsCRP

**Table S6** Incremental value of changes in TyG-WC for incident CVD

|  | **C-statistic^a^** | **C-statistic^b^** | **ΔC-statistic^c^** |
| --- | --- | --- | --- |
| Changes in TyG-WC vs. Baseline WC | 0.584(0.561-0.608) | 0.564(0.540-0.588) | 0.021(0.005-0.037) |
| Changes in TyG-WC vs. Baseline TG | 0.584(0.561-0.608) | 0.539(0.516-0.563) | 0.045(0.023-0.066) |
| Changes in TyG-WC vs. Baseline FBG | 0.584(0.561-0.608) | 0.543(0.519-0.578) | 0.041(0.014-0.070) |
| Changes in TyG-WC vs. Baseline TyG-WC | 0.584(0.561-0.608) | 0.570(0.547-0.594) | 0.014(0.003-0.025) |

Baseline WC, TG, FBG, and TyG-WC were assessed as quartiles.

WC: waist circumference; TyG: triglyceride‑glucose index; TG: triglyceride; FBG: fasting plasma glucose.

^a^C-statistic of changes in TyG-WC.

^b^C-statistic of baseline WC, TG, FBG, or TyG-WC.

^c^ΔC-statistic = C-statistic^a^ - C-statistic^b^.

**Table S7** Incremental value of changes in TyG-BMI for incident CVD

|  | **C-statistic^a^** | **C-statistic^b^** | **ΔC-statistic^c^** |
| --- | --- | --- | --- |
| Changes in TyG-BMI vs. Baseline BMI | 0.565(0.541-0.588) | 0.552(0.528-0.577) | 0.012(-0.002-0.025) |
| Changes in TyG-BMI vs. Baseline TG | 0.565(0.541-0.588) | 0.539(0.516-0.563) | 0.025(0.000-0.048) |
| Changes in TyG-BMI vs. Baseline FBG | 0.565(0.541-0.588) | 0.543(0.519-0.568) | 0.021(-0.008-0.051) |
| Changes in TyG-BMI vs. Baseline TyG-BMI | 0.565(0.541-0.588) | 0.566(0.542-0.590) | -0.002(-0.013-0.009) |

Baseline BMI, TG, FBG, and TyG-BMI were assessed as quartiles.

BMI: body mass index; TyG: triglyceride‑glucose index; TG: triglyceride; FBG: fasting plasma glucose.

^a^C-statistic of changes in TyG-BMI.

^b^C-statistic of baseline BMI, TG, FBG, or TyG-BMI.

^c^ΔC-statistic = C-statistic^a^ - C-statistic^b^.

**Table S8** Incremental value of changes in TyG-WHtR for incident CVD

|  | **C-statistic^a^** | **C-statistic^b^** | **ΔC-statistic^c^** |
| --- | --- | --- | --- |
| Changes in TyG-WHtR vs. Baseline WHtR | 0.578(0.554-0.601) | 0.567 (0.543-0.591) | 0.010(-0.004-0.025) |
| Changes in TyG-WHtR vs. Baseline TG | 0.578(0.554-0.601) | 0.539(0.516-0.563) | 0.038(0.014-0.061) |
| Changes in TyG-WHtR vs. Baseline FBG | 0.578(0.554-0.601) | 0.543(0.519-0.568) | 0.034(0.005-0.064) |
| Changes in TyG-WHtR vs. Baseline TyG-WHtR | 0.578(0.554-0.601) | 0.571(0.547-0.594) | 0.007(-0.007-0.020) |

Baseline WHtR, TG, FBG, and TyG-WHtR were assessed as quartiles.

WHtR: waist height ratio; TyG: triglyceride‑glucose index; TG: triglyceride; FBG: fasting plasma glucose.

^a^C-statistic of changes in TyG-WHtR.

^b^C-statistic of baseline WHtR, TG, FBG, or TyG-WHtR.

^c^ΔC-statistic = C-statistic^a^ - C-statistic^b^.

**Table S9** Associations of changes in TyG-WC and cumulative TyG-WC with cardiovascular disease incidence in complete dataset

|  | **Model 1^a^** |  | **Model 2^b^** |  | **Model 3^c^** |  | **Model 4^d^** |  |
| --- | --- | --- | --- | --- | --- | --- | --- | --- |
|  | **OR (95%CI)** | **P value** | **OR (95%CI)** | **P value** | **OR (95%CI)** | **P value** | **OR (95%CI)** | **P value** |
| **Changes in TyG-WC** |  |  |  |  |  |  |  |  |
| Class1 | Reference |  | Reference |  | Reference |  | Reference |  |
| Class2 | 1.57 (1.22~2.03) | <0.001 | 1.57 (1.22~2.03) | 0.001 | 1.4 (1.08~1.83) | 0.012 | 1.36 (1.04~1.79) | 0.025 |
| Class3 | 1.94 (1.49~2.52) | <0.001 | 1.95 (1.5~2.54) | <0.001 | 1.54 (1.15~2.05) | 0.003 | 1.51 (1.11~2.04) | 0.008 |
| Class4 | 2.79 (2.07~3.74) | <0.001 | 2.78 (2.06~3.75) | <0.001 | 1.89 (1.33~2.68) | <0.001 | 1.89 (1.29~2.76) | 0.001 |
| **Cumulative TyG-WC** |  |  |  |  |  |  |  |  |
| Quartile1 | Reference |  | Reference |  | Reference |  | Reference |  |
| Quartile2 | 1.47 (1.12~1.94) | 0.006 | 1.47 (1.12~1.94) | 0.006 | 1.37 (1.03~1.81) | 0.03 | 1.35 (1.01~1.80) | 0.041 |
| Quartile3 | 1.75 (1.34~2.29) | <0.001 | 1.75 (1.34~2.3) | <0.001 | 1.42 (1.06~1.9) | 0.018 | 1.37 (1.01~1.85) | 0.042 |
| Quartile4 | 2.48 (1.91~3.21) | <0.001 | 2.49 (1.91~3.24) | <0.001 | 1.81 (1.34~2.46) | <0.001 | 1.81 (1.30~2.53) | <0.001 |
| P for trend | 1.33 (1.23~1.45) | <0.001 | 1.34 (1.23~1.45) | <0.001 | 1.2 (1.09~1.32) | <0.001 | 1.20 (1.08~1.33) | 0.001 |

WC: waist circumference; CI: confidence interval; OR: odds ratio; TyG: triglyceride-glucose index

a Adjusted for age and sex

b Adjusted for age, sex, marital status, educational level, smoking status, and drinking status

c Adjusted for variables in Model 2 and history of hypertension, diabetes, dyslipidaemia, kidney disease, medication use for hypertension, medication use for diabetes, medication use for dyslipidaemia, systolic blood pressure, diastolic blood pressure

d Adjusted for variables in Model 3 and total cholesterol, HDL-C, LDL-C, HbA1c, the eGFR, and hsCRP

**Table S10** Associations of changes in TyG-BMI and cumulative TyG-BMI with cardiovascular disease incidence in complete dataset

|  | **Model 1^a^** |  | **Model 2^b^** |  | **Model 3^c^** |  | **Model 4^d^** |  |
| --- | --- | --- | --- | --- | --- | --- | --- | --- |
|  | **OR (95%CI)** | **P value** | **OR (95%CI)** | **P value** | **OR (95%CI)** | **P value** | **OR (95%CI)** | **P value** |
| **Changes in TyG-BMI** |  |  |  |  |  |  |  |  |
| Class1 | Reference |  | Reference |  | Reference |  | Reference |  |
| Class2 | 1.53 (1.21~1.93) | <0.001 | 1.54 (1.21~1.95) | <0.001 | 1.3 (1.01~1.66) | 0.039 | 1.26 (0.98~1.62) | 0.071 |
| Class3 | 1.73 (1.34~2.23) | <0.001 | 1.73 (1.33~2.25) | <0.001 | 1.28 (0.96~1.71) | 0.091 | 1.24 (0.91~1.67) | 0.173 |
| Class4 | 2.65 (1.94~3.63) | <0.001 | 2.67 (1.95~3.67) | <0.001 | 1.6 (1.11~2.3) | 0.013 | 1.60 (1.08~2.36) | 0.019 |
| **Cumulative TyG-BMI** |  |  |  |  |  |  |  |  |
| Quartile1 | Reference |  | Reference |  | Reference |  | Reference |  |
| Quartile2 | 1.47 (1.12~1.94) | 0.006 | 1.47 (1.12~1.93) | 0.005 | 1.31 (1~1.73) | 0.052 | 1.28 (0.96~1.69) | 0.088 |
| Quartile3 | 1.75 (1.34~2.29) | <0.001 | 1.57 (1.2~2.06) | 0.001 | 1.23 (0.92~1.64) | 0.167 | 1.20 (0.88~1.62) | 0.244 |
| Quartile4 | 2.48 (1.91~3.21) | <0.001 | 2.29 (1.75~2.98) | <0.001 | 1.53 (1.13~2.08) | 0.006 | 1.52 (1.09~2.12) | 0.014 |
| P for trend | 1.33 (1.23~1.45) | <0.001 | 1.29 (1.19~1.4) | <0.001 | 1.13 (1.02~1.24) | 0.016 | 1.13 (1.01~1.25) | 0.030 |

BMI: body mass index; CI: confidence interval; OR: odds ratio; TyG: triglyceride-glucose index

a Adjusted for age and sex

b Adjusted for age, sex, marital status, educational level, smoking status, and drinking status

c Adjusted for variables in Model 2 and history of hypertension, diabetes, dyslipidaemia, kidney disease, medication use for hypertension, medication use for diabetes, medication use for dyslipidaemia, systolic blood pressure, diastolic blood pressure

d Adjusted for variables in Model 3 and total cholesterol, HDL-C, LDL-C, HbA1c, the eGFR, and hsCRP

**Table S11** Associations of changes in TyG-WHtR and cumulative TyG-WHtR with cardiovascular disease incidence in complete dataset

|  | **Model 1^a^** |  | **Model 2^b^** |  | **Model 3^c^** |  | **Model 4^d^** |  |
| --- | --- | --- | --- | --- | --- | --- | --- | --- |
|  | **OR (95%CI)** | **P value** | **OR (95%CI)** | **P value** | **OR (95%CI)** | **P value** | **OR (95%CI)** | **P value** |
| **Changes in TyG-WHtR** |  |  |  |  |  |  |  |  |
| Class1 | Reference |  | Reference |  | Reference |  | Reference |  |
| Class2 | 1.46 (1.14~1.86) | 0.003 | 1.45 (1.13~1.86) | 0.003 | 1.24 (0.96~1.6) | 0.104 | 1.19 (0.91~1.55) | 0.195 |
| Class3 | 1.74 (1.34~2.26) | <0.001 | 1.75 (1.34~2.27) | <0.001 | 1.3 (0.97~1.73) | 0.078 | 1.23 (0.91~1.67) | 0.182 |
| Class4 | 2.45 (1.81~3.31) | <0.001 | 2.42 (1.79~3.27) | <0.001 | 1.52 (1.07~2.18) | 0.021 | 1.49 (1.01~2.20) | 0.046 |
| **Cumulative TyG-WHtR** |  |  |  |  |  |  |  |  |
| Quartile1 | Reference |  | Reference |  | Reference |  | Reference |  |
| Quartile2 | 1.43 (1.09~1.88) | 0.01 | 1.42 (1.08~1.87) | 0.013 | 1.23 (0.93~1.64) | 0.147 | 1.19 (0.89~1.59) | 0.229 |
| Quartile3 | 1.68 (1.28~2.21) | <0.001 | 1.65 (1.26~2.17) | <0.001 | 1.34 (1~1.79) | 0.05 | 1.27 (0.94~1.73) | 0.119 |
| Quartile4 | 2.26 (1.72~2.95) | <0.001 | 2.25 (1.71~2.95) | <0.001 | 1.53 (1.11~2.1) | 0.009 | 1.47 (1.05~2.07) | 0.027 |
| P for trend | 1.29 (1.19~1.41) | <0.001 | 1.29 (1.19~1.41) | <0.001 | 1.14 (1.03~1.26) | 0.01 | 1.13 (1.01~1.26) | 0.029 |

WHtR: waist height ratio; CI: confidence interval; OR: odds ratio; TyG: triglyceride-glucose index

a Adjusted for age and sex

b Adjusted for age, sex, marital status, educational level, smoking status, and drinking status

c Adjusted for variables in Model 2 and history of hypertension, diabetes, dyslipidaemia, kidney disease, medication use for hypertension, medication use for diabetes, medication use for dyslipidaemia, systolic blood pressure, diastolic blood pressure

d Adjusted for variables in Model 3 and total cholesterol, HDL-C, LDL-C, HbA1c, the eGFR, and hsCRP

**K-means clustering**

**The principle of the K-means clustering algorithm**

As the most commonly used clustering algorithm, K-means is an unsupervised machine learning method that categorizes a dataset into K classes by minimizing the sum of squared Euclidean distance (SSED) within each class[1]. The working procedure of this algorithm can be summarized in three steps: first, K participants were randomly selected as the initial class centres; subsequently, the remaining participants were assigned to the nearest centres; last, we updated K centres based on newly established classes. After several iterations of repeating the above procedures, the SSED within each class was minimized, resulting in the final division of participants into K classes[2]. To identify the optimal number of classes, we visually analysed the decrease in the SSED resulting from the variation in the number of classes. We ultimately determined the number of classes, K, to be 4, as the reduction in SSED became negligible with more than 4 classes.

**The definition of changes in TyG-related indices**

Changes in TyG-WC was classified into 4 classes by the K-means algorithm, and the paired Wilcoxon test was used to assess the difference of TyG-WC at Wave 1 and Wave 3 within each class. For Class 1 (n = 825), the TyG-WC change pattern presented consistent low TyG-WC (612.01 ± 46.65 in Wave 1, 611.21 ± 49.80 in Wave 3, *P* = 0.95); for Class 2 (n = 1,106), the TyG-WC ranged from 705.25 ± 47.28 in Wave 1 to 716.15 ± 47.03 in Wave 3 (*P* < 0.001), indicating a moderate TyG-WC with a slight increasing trend; for Class 3 (n = 882), the TyG-WC ranged from 804.38 ± 50.74 in Wave 1 to 816.65 ± 51.72 in Wave 3 (*P* < 0.001), representing a high TyG-WC with a slight increasing trend; for Class 4 (n = 430), the change pattern presented consistent highest TyG-WC (934.77 ± 73.38 in Wave 1, 939.72 ± 69.97 in Wave 3, *P* = 0.083) (Fig.  2B). The distribution of TyG-WC according to the classes of TyG-WC change is shown in Fig. 2C, D.

Similarly, changes in TyG-BMI was classified into 4 classes (Fig. S1). For Class 1 (n = 940), the TyG-BMI change pattern presented consistent low TyG-BMI (164.06 ± 14.88 in Wave 1, 163.93 ± 14.80 in Wave 3, *P* = 0.52); for Class 2 (n = 1,211), the TyG-BMI ranged from 197.18 ± 14.05 in Wave 1 to 200.87 ± 14.06 in Wave 3 (*P* < 0.001), representing a moderate TyG-BMI with a slight increasing trend; for Class 3 (n = 781), the TyG-BMI ranged from 232.88 ± 17.10 in Wave 1 to 236.00 ± 16.59 in Wave 3 (*P* < 0.001), representing a high TyG-BMI with a slight increasing trend; for Class 4 (n = 311), the change pattern presented consistent highest TyG-BMI (280.65 ± 34.13 in Wave 1, 277.40 ± 25.57 in Wave 3, *P* = 0.84).

The clustering process of TyG-WHtR was similar (Fig. S2). For Class 1, TyG-WHtR change pattern presented consistent low TyG-WHtR (3.89 ± 0.31 in Wave 1, 3.88 ± 0.32 in Wave 3, *P* = 0.94); for Class 2 (n = 1,106), the TyG-WHtR ranged from 4.52 ± 0.29 in Wave 1 to 4.62 ± 0.31 in Wave 3 (*P* < 0.001), representing a moderate TyG-WHtR with a slight increasing trend; for Class 3 (n = 882), the TyG-WHtR ranged from 5.16 ± 0.32 in Wave 1 to 5.27 ± 0.34 in Wave 3 (*P* < 0.001), representing a high TyG-WHtR with a slight increasing trend; for Class 4 (n = 430), the change pattern presented consistent highest TyG-WHtR (6.00 ± 0.45 in Wave 1, 6.02 ± 0.46 in Wave 3, *P* = 0.31).

**eReference**

1. Sinaga KP, Yang M-S. Unsupervised K-Means Clustering Algorithm. IEEE Access. 2020;8:80716–27.

2. Singh A, Yadav A, Rana A. K-means with Three different Distance Metrics. IJCA. 2013;67:13–7.


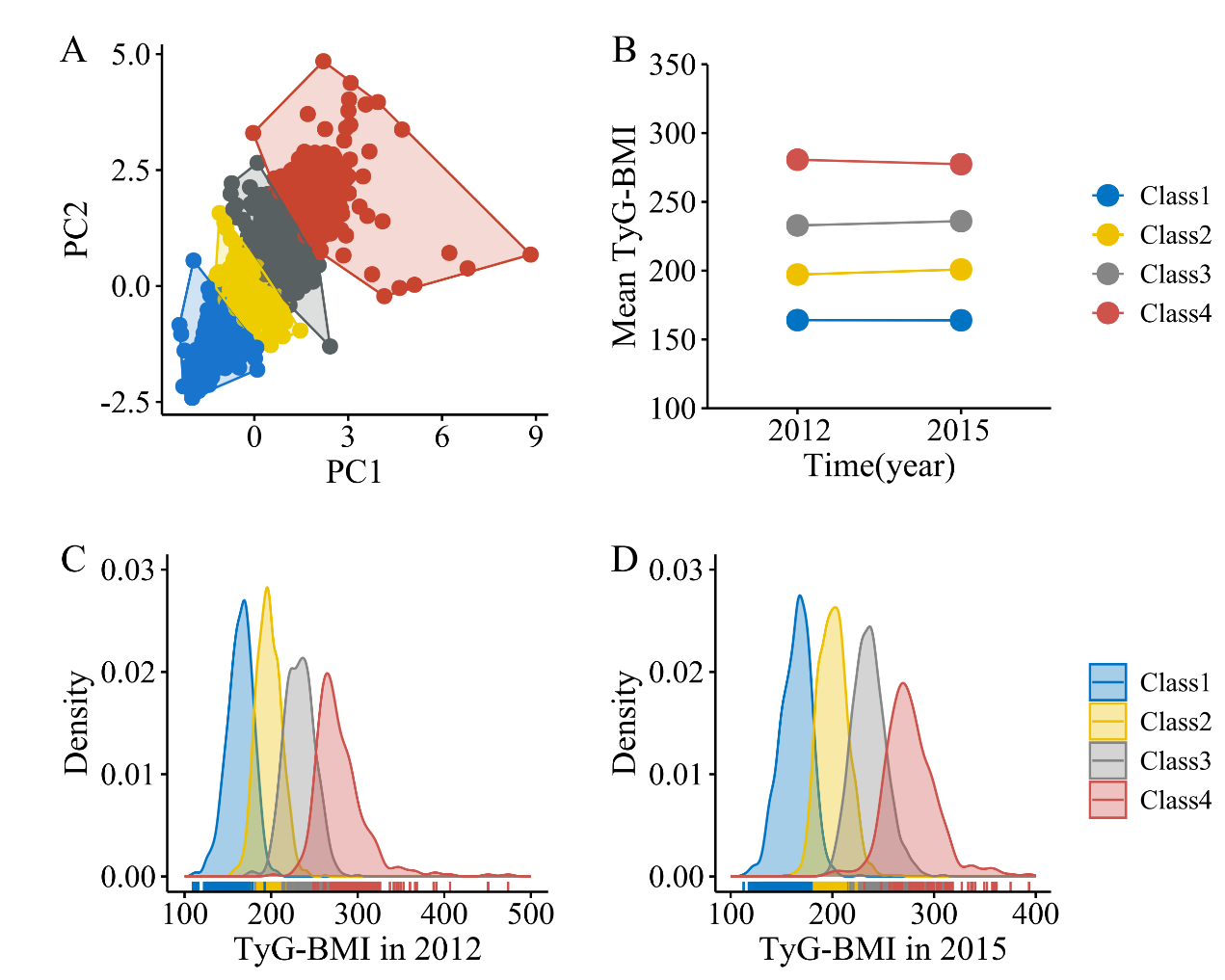


**Fig. S1** Clustering of changes in TyG-BMI from Wave 1 to Wave 3. Change in TyG-BMI classified into four classes using the K‑means algorithm **(A)**; Mean TyG-BMI for the four classes in 2012 and 2015 **(B)**; Distribution of TyG-BMI in 2012 or 2015 **(C, D)**. PC: principal component; TyG: triglyceride‑glucose; BMI: body mass index.


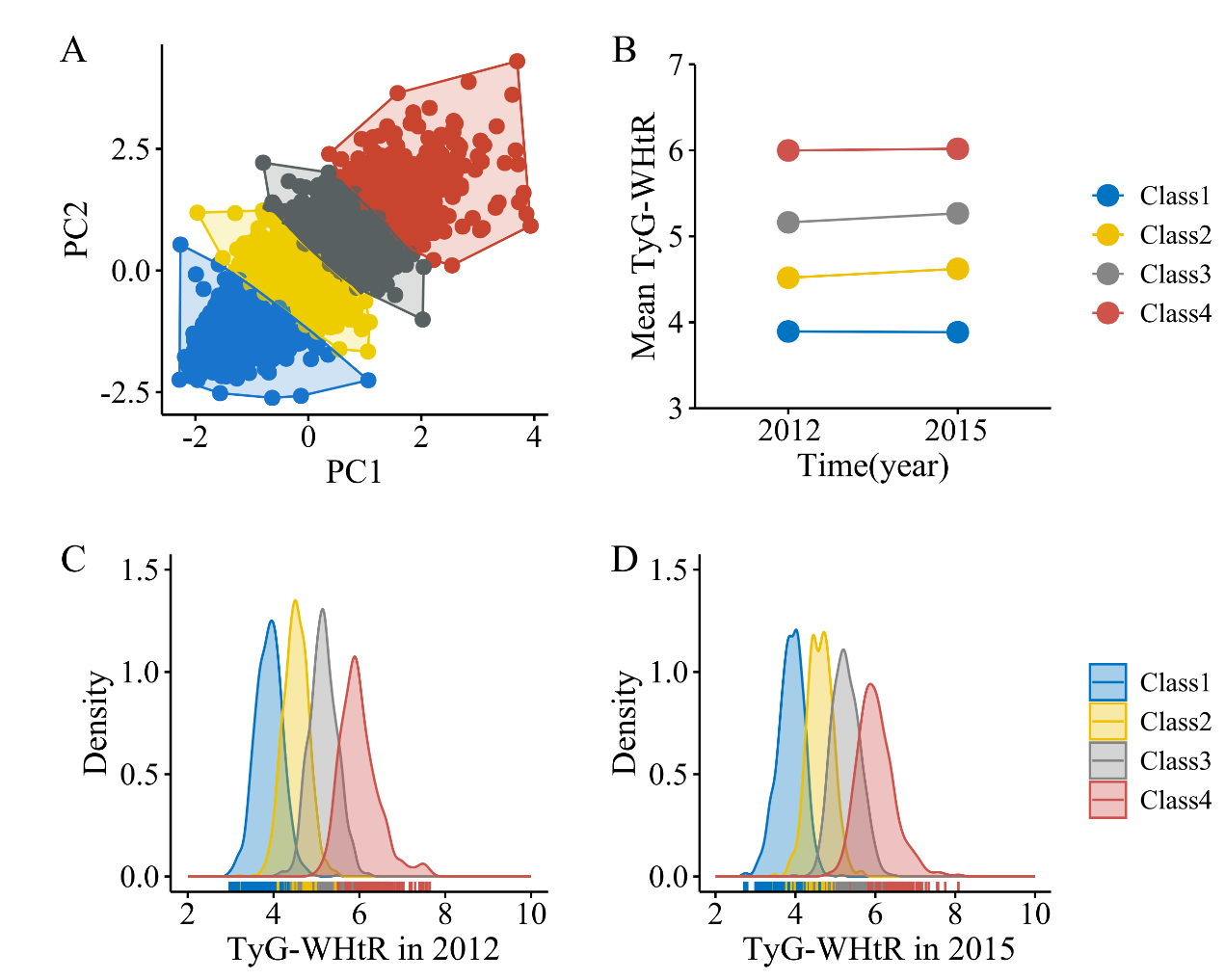


**Fig. S2** Clustering of changes in TyG-WHtR from Wave 1 to Wave 3. Change in TyG-WHtR classified into four classes using the K‑means algorithm **(A)**; Mean TyG-WHtR for the four classes in 2012 and 2015 **(B)**; Distribution of TyG-WHtR in 2012 or 2015 **(C, D)**. PC: principal component; TyG: triglyceride‑glucose; WHtR: waist height ratio.
